# Copper-catalyzed asymmetric sp^3^ C–H arylation of tetrahydroisoquinoline mediated by a visible light photoredox catalyst

**DOI:** 10.3762/bjoc.12.260

**Published:** 2016-12-06

**Authors:** Pierre Querard, Inna Perepichka, Eli Zysman-Colman, Chao-Jun Li

**Affiliations:** 1 Department of Chemistry, FQRNT Center for Green Chemistry and Catalysis, McGill University, 801 Sherbrooke Street West, Montreal, Quebec H3A 0B8, Canada; 2 Organic Semiconductor Centre, EaStCHEM School of Chemistry, University of St Andrews, St Andrews, Fife, KY16 9ST, UK

**Keywords:** C–H arylation, copper catalyst, enantioselectivity, visible light

## Abstract

This report describes a highly enantioselective oxidative sp^3^ C–H arylation of *N*-aryltetrahydroisoquinolines (THIQs) through a dual catalysis platform. The combination of the photoredox catalyst, [Ir(ppy)_2_(dtbbpy)]PF_6_, and chiral copper catalysts provide a mild and highly effective sp^3^ C–H asymmetric arylation of THIQs.

## Introduction

Functionalization of sp^3^ C–H bonds is a unique and powerful transformation in modern organic synthesis, which remains a challenging process despite the advances that have been made in this field [1]. The directing group strategies are widely used and developed to achieve enantioselective metal-catalyzed C–H bond functionalizations in recent years. Unactivated alkyl C–H bond activation (i.e., without any directing group) is of great interest in terms of atom economy, nevertheless enantioselectivity is difficult to control due to often-required harsh reaction conditions. Therefore, the development of simple and facile processes to functionalize sp^3^ C–H bonds under mild conditions in the absence of directing groups is of great interest [2].

The emerging and expanding field of visible-light-mediated photoredox catalysis presents unique opportunities for the conception of new synthetic routes [3–12]. Upon exposure to visible light, photoredox catalysts can function as both reductant and oxidant, thereby providing extremely important tools for potential transition-metal-catalyzed enantioselective reactions of sp^3^ C–H bonds, which could be carried out at low temperature and under mild reaction conditions [13–14]. We envisioned that combining photoredox catalysis with typical cross-coupling methods will allow us to design a visible-light-mediated photoredox asymmetric arylation of tetrahydroisoquinolines (THIQs) [15–20].

**Scheme 1:**
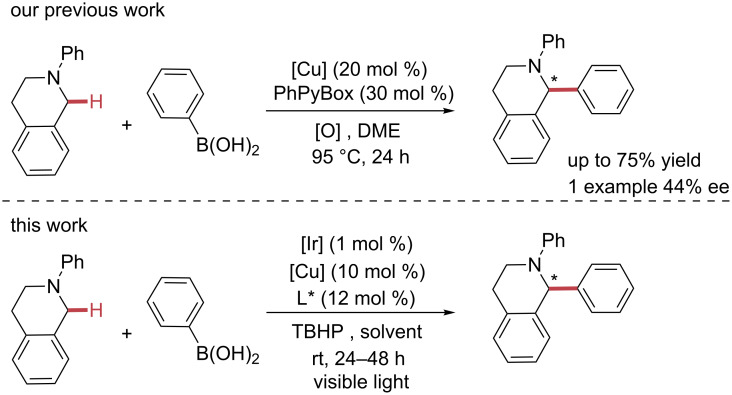
Design light-mediated arylation of THIQs.

During the last decade, numerous examples of sp^3^ C–H bond arylation procedures have been developed [1,21–29]. In 2008, our group developed the first direct sp*^3^* C–H arylation of THIQ with arylboronic acids using a copper catalyst (Scheme 1) [30]. Oxygen gas and *tert*-butyl hydroperoxide (TBHP) were used as external oxidants, which gave moderate to good isolated yields (up to 75%). In addition, we demonstrated the first enantioselective arylation of THIQ using phenylboronic acid with 44% enantiomeric excess (ee), but very poor yield of the optically active products. Lowering the reaction temperature, in order to increase the corresponding ee, resulted in inhibition of the reaction.

More recently, Liu et al. have demonstrated the arylation of THIQs with arylboronic esters via asymmetric organocatalysis methodology [25,28]. The use of chiral tartaric acid derivatives, 2,3-dichloro-5,6-dicyano-1,4-benzoquinone (DDQ) and high temperature (70 °C) were found to be the optimal conditions to obtain the desired arylated product with acceptable yield and good enantioselectivity. However, this methodology has shown limitations in terms of substrate scope: only phenylboronic esters with electron-donating substituents yielded the corresponding products.

We herein report the first visible light-mediated asymmetric cross-coupling arylation of sp*^3^* C–H bonds adjacent to nitrogen, combining photoredox catalysis with metal-catalyzed transformations.

## Results and Discussion

### Optimisation of reaction conditions

In our previous work on arylation of *N-*aryltetrahydroisoquinoline [30], we demonstrated that lowering the temperature from 90 °C to room temperature in the reaction with copper(I) bromide caused a significant drop in yield. During optimisation of the reaction system, TBHP was found to be the best external oxidant for this reaction over many others [31]. To accelerate the reaction at lower temperature, we reasoned that a light-mediated photoredox system might help, which indeed has improved the reaction yield and enantioselectivity. Different iridium and ruthenium photoredox catalysts were evaluated and [Ir(ppy)_2_(d*t*bbpy)]PF_6_ was found to be the most efficient [32]. With this iridium photoredox catalyst, TBHP, and copper(I) bromide co-catalyst in DME as solvent, we observed a trace amount of the desired product at room temperature. When different copper salts were evaluated, it was found that CuBr was less active (Table 1, entry 1) and copper(II) bromide provided the highest yield for the arylation of THIQ with phenylboronic acid (**2**, Table 1, entry 2). Other copper salts such as Cu(OTf)_2_ and Cu(OAc)_2_ were much less effective (Table 1, entries 3 and 4). A significant increase of yield was observed when the stoichiometry of the system was changed to a slight excess of arylboronic acid. When more than 1.6 equivalents of **2** were involved in the reaction, a drastic acceleration of the reaction was observed, leading to up to 85% yield (Table 1, entries 5 and 6). During the investigation of solvent influence on the formation of **3a**, it was found that polar solvents such as DCE gave the best yields, compared to less polar solvents such as toluene and THF (Table 1, entries 7 and 8). On the other hand, highly polar solvents such as MeCN and MeOH were not beneficial for the formation of the desired product **3a** (Table 1, entries 9 and 10). Control experiments performed in the absence of photoredox catalyst and/or transition metal copper(II) salt (Table 1, entries 11–13) showed very poor reactivity. Moreover, in the absence of light, an extremely poor yield was obtained (Table 1, entry 14).

**Table 1:** Optimization of reaction conditions^a^.

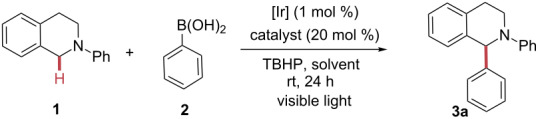				

Entry	Catalyst	Solvent	**2** (equiv)	Yield of **3a** (%)

1	CuBr	DCE	1.6	19
2	CuBr_2_	DCE	1.6	29
3	Cu(OTf)_2_	DCE	1.6	2
4	Cu(OAc)_2_	DCE	1.6	14
5	CuBr_2_	DCE	2	72
6	CuBr_2_	DCE	3	**85**
7	CuBr_2_	THF	3	15
8	CuBr_2_	toluene	3	23
9	CuBr_2_	MeCN	3	11
10	CuBr_2_	MeOH	3	13
11^b^	CuBr_2_	DCE	3	12
12^b^	–	DCE	3	0
13^b,c^	CuBr_2_	DCE	3	0
14^d^	CuBr_2_	DCE	3	12

^a^Reaction conditions: THIQs (0.10 mmol), arylboronic acid (0.30 mmol), TBHP (0.16 mmol), [Ir(ppy)_2_(dtbbpy)]PF_6_ (0.001 mmol), CuBr_2_ (0.02 mmol), DCE (0.5 mL), under argon atmosphere. NMR yields are reported. ^b^Reaction carried out without Ir(III) photoredox catalyst. ^c^Reaction carried out without TBHP. ^d^Reaction performed in absence of light. All reported yields were determined by ^1^H NMR spectroscopy using dibromomethane as an internal standard.

### General scope of reaction

**Figure 1:**
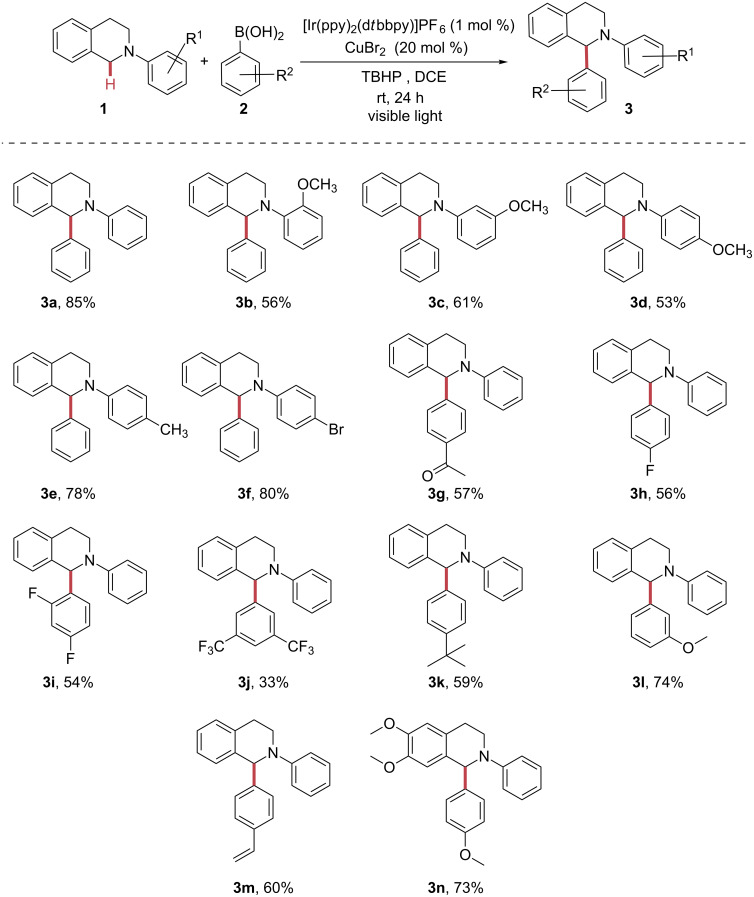
Reaction scope. Reaction conditions: THIQs (0.10 mmol), arylboronic acid (0.30 mmol), TBHP (0.2 mmol), [Ir(ppy)_2_(d*t*bbpy)]PF_6_ (0.001 mmol), CuBr_2_ (0.02 mmol), DCE (0.5 mL), under argon atmosphere.

With the optimized reaction conditions in hand, the substrate scope was investigated (Figure 1). *N*-Phenyltetrahydroisoquinoline (**1**) combined with phenylboronic acid (**2**) gave rise to 85% yield of the corresponding arylated product **3a**. *N*-Phenyl-substituted THIQs bearing electron-donating groups (EDG), such a OMe and Me, were tolerated in our reaction system. We were surprised to see that strong electron-donating substituents such as OMe gave lower yields (**3b**, **3c** and **3d**), which we attribute to the lowered oxidation potentials of the tertiary amine, favouring side reactions. It is notable that weaker EDG substituents on the aryl moiety (e.g., Me) resulted in higher yields (**3e**). Electron-withdrawing groups (EWG) such as Br were tolerated and yielded the desired product in 80% yield (**3f**). Aromatic boronic acids possessing both electron-withdrawing and electron-donating substituents were evaluated under our reaction conditions and all resulted in good yields. While aromatic boronic acids substituted with electron-withdrawing groups (e.g., acyl, F or CF_3_) were likewise tolerated well (**3g**–**j**). Aromatic boronic acids substituted with electron-donating groups resulted in the formation of the corresponding arylated products with higher yields (**3k**–**n**).

### Enantioselective arylation reaction

**Scheme 2:**
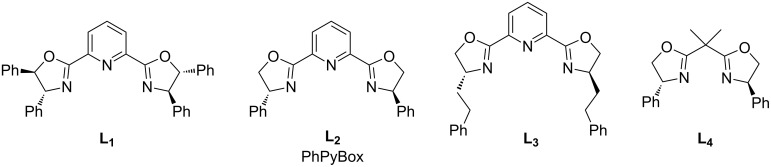
Evaluation of chiral ligands.

Subsequently, we explored the asymmetric version of this arylation reaction with various chiral ligands (see Scheme 2 and Supporting Information File 1, Table S3, for a detailed screening table). Among them, Box-type ligands have demonstrated a good performance in this reaction, affording low to good enantioselectivities.

We began our study by evaluating the efficiency of the ligands using the standard arylation of THIQ with phenylboronic acid. A modest enantiomeric ratio (er) of the C−H coupling reaction was obtained using **L_1_** ligand (Table 2, entry 1) at low temperature (4 °C). On the other hand, the commercially available mono-arylated PyBox **L_2_** gave very good er under our reaction conditions (Table 2, entry 2). It is noteworthy that the er observed was higher when copper(I) bromide was used as a co–catalyst, compared to copper(II) bromide (Table 2, entry 3), possibly due to the Lewis acidity difference of Cu(I) and Cu(II). However, the yield of the desired optically active product **3a** dropped by about half, when CuBr was used as catalyst. Alkyl-substituted PyBox such as **L_3_** was not beneficial to the enantioselectivity (Table 2, entry 4). The efficacy of N,N-Box ligand (**L_4_**) was investigated and it appeared that the pyridine motif was extremely important to achieve high enantioselectivity (Table 2, entry 5).

**Table 2:** Effect of chiral ligand on the enantioselectivity of coupling of *N*-phenyltetrahydroisoquinoline with phenylboronic acid^a^.

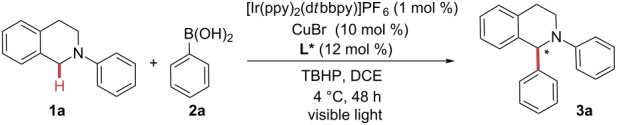		

Entry	L*	er

1	**L_1_**	69:31
2	**L_2_**	82:18
3^b^	**L_2_**	68:32
4	**L_3_**	54:46
5	**L_4_**	54:46

^a^Reaction conditions: THIQs (0.10 mmol), arylboronic acid (0.30 mmol), TBHP (0.2 mmol), [Ir(ppy)_2_(dtbbpy)]PF_6_ (0.001 mmol), CuBr (0.01 mmol), L* (0.012 mmol), DCE (0.5 mL), under argon atmosphere. ^b^CuBr_2_ was used. All reported enantiomeric ratios were determined using a Chiralcel OD-H column and 96:4 hexane/isopropanol as an eluent (Supporting Information File 1).

To evaluate the scope of the enantiomeric selectivity of the arylation reaction, copper(I) bromide together with (*R*,*R*)-PhPyBox **L_2_** at 4 °C was used as the standard conditions. We were pleased to see that our model reaction yielded **3a** with good enantiomeric ratio (Table 3, entry 1). In the presence of the other enantiomer of **L_2_**, (*S*,*S*)-PhPyBox, the reaction afforded good er. When *N*-(2-methhoxyphenyl)tetrahydroisoquinoline was used, the corresponding enantiomer was obtained with similar enantioselectivity (Table 3, entry 2). *N*-Aryl-substituted THIQs gave high er, when either EDG or EWG were present (Table 3, entries 3–6). High and moderate enantiomeric ratios were obtained, respectively, when vinyl-substituted arylboronic acids and fluoro-substituted arylboronic acids were subjected to the reaction system (Table 3, entries 7 and 8).

**Table 3:** Enantioselective arylation reaction^a^.

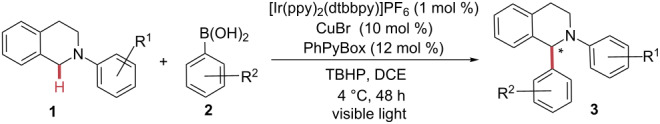				

Entry	Product	R^1^	R^2^	er

1	**3a**	H	H	19:81
2^b^	**3b**	2-OMe	H	84:16
3	**3c**	3-OMe	H	10:90
4	**3d**	4-OMe	H	15:85
5	**3e**	4-Me	H	24:76
6	**3f**	4-Br	H	19:81
7	**3m**	H	4-vinyl	19:81
8	**3j**	H	2,4-difluoro	37:63

^a^Reaction conditions: THIQs (0.10 mmol), arylboronic acid (0.30 mmol), TBHP (0.2 mmol), [Ir(ppy)_2_(dtbbpy)]PF_6_ (0.001 mmol), CuBr (0.01 mmol), (*R*,*R*)-2,6-Bis(4-phenyl-2-oxazolinyl)pyridine (0.012 mmol), DCE (0.5 mL), under argon atmosphere. ^b^(*S*,*S*)-2,6-bis(4-phenyl-2-oxazolinyl)pyridine was used instead. All reported yields enantiomeric ratios were determined using a Chiralcel OD-H column and 96:4 hexane/isopropanol as an eluent (Supporting Information File 1).

**Scheme 3:**
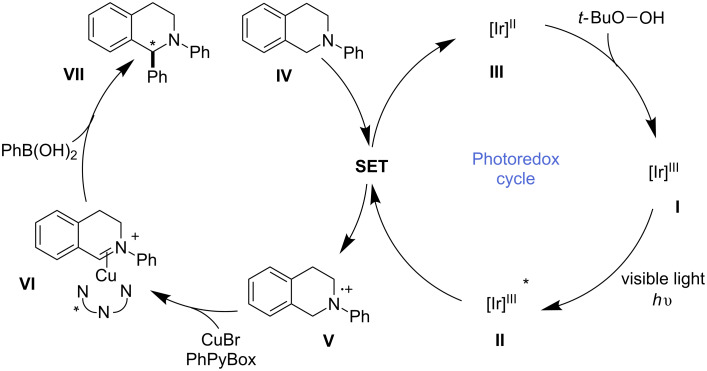
Proposed reaction mechanism.

A tentative reaction mechanism has been proposed in Scheme 3, in order to rationalize this arylation reaction. Upon visible light irradiation, [Ir(ppy)_2_(dtbbpy)]PF_6_ **I** was converted into an excited state **II**, Ir(III)* [11,33–37]. The THIQ undergoes a single electron transfer (SET), reducing the iridium complex to Ir(II) **III** and oxidizing the the nitrogen of THIQ **IV** to its radical cation **V**, which then undergoes a hydride abstraction to form the iminium salt form **VI**, of the THIQ. The pre-formed chiral PhCu–PyBox complex [38], coordinates to the iminium cation **VI**, followed by stereofacial nucleophilic addition of the arylboronic acid to produce the desired enantioenriched arylated product **VII**. The Ir(III) is regenerated in the presence of the sacrificial external oxidant TBHP.

## Conclusion

In conclusion, we have successfully developed a highly efficient light-mediated coupling method for the direct asymmetric arylation of *N*-arylated tetrahydroisoquinolines (THIQs) with arylboronic acids. Using [Ir(ppy)_2_(dtbbpy)]PF_6_ as photoredox catalyst provided a novel facile method to build important arylated compounds in very high yields under very mild conditions. The combination of copper salts and PhPyBox as chiral ligand have demonstrated its efficiency producing good enantioselectivity and tolerated a fairly diverse substrate scope. We envisioned that this visible light-mediated asymmetric arylation reaction could be extended to other sp^3^ C–H bonds. The development of new light-mediated processes for stereoselective functionalization of unactivated C−H bonds is currently undergoing in our laboratory.

## Experimental

**General procedure for the sp^3^ C–H arylation of THIQs with boronic acid derivatives** (Figure 1). A V-shaped 10 mL Biotage reaction vial was charged with [Ir(ppy)_2_(dtbbpy)]PF_6_ (1 mol %, 1.0 mg), CuBr_2_ (10 mol %, 2.23 mg), *N*-phenyltetrahydroisoquinoline (0.1 mmol), and the corresponding phenylboronic acid (0.3 mmol), evacuated and refilled with argon three times. DCE (0.5 mL) was added, followed by subsequent slow addition of TBHP (0.16 mmol). The reaction vessel was sealed, placed under white light bulbs irradiation with vigorous stirring (approx. 1000 rpm) and hold for 24 h. The mixture was diluted with ethyl acetate (2 mL), washed with water (2 mL), filtered through a pad of silica, and rinsed with additional ethyl acetate. The combined organic phase was concentrated and purified by column chromatography or preparative thin-layer chromatography on silica gel to yield the corresponding arylated compound **3**. Dibromomethane was used as internal standard for ^1^H NMR analysis.

**Variation for enantioselective sp^3^ C–H arylation of THIQs with boronic acid derivatives** (Table 3). A V-shaped 10 mL Biotage reaction vial was charged with CuBr (10 mol %, 1.43 mg) and PhPybox (12 mol %, 4.43 mg), evacuated and refilled with argon three times, and then 0.1 mL of DCE was added. The reaction was stirred for 30 min. *N*-Phenyltetrahydroisoquinoline (0.1 mmol), [Ir(ppy)_2_(dtbbpy)]PF_6_ (1 mol %, 1.0 mg) and the corresponding phenylboronic acid (0.3 mmol) were added, and then the atmosphere was evacuated and refilled with argon three times. DCE (0.4 mL) was added followed by subsequent slow addition of TBHP (0.16 mmol). The reaction vessel was sealed, placed under white light bulbs irradiation with vigorous stirring (approx. 1000 rpm) and held for 48 h in a cold room (4 °C). The mixture was diluted with ethyl acetate (2 mL), washed with water (2 mL), filtered through a pad of silica, and rinsed with additional ethyl acetate. The combined organic phase was concentrated and purified by column chromatography or preparative thin-layer chromatography on silica gel to yield the corresponding arylated compound **3**. Dibromomethane was used as internal standard for ^1^H NMR analysis.

## Supporting Information

File 1 Experimental and copies of spectra.
