# Supplementary material for: Copper-catalyzed asymmetric sp3 C–H arylation of tetrahydroisoquinoline mediated by a visible light photoredox catalyst
Source: Beilstein J Org Chem. 2016 Dec 6;12:2636–43. doi: 10.3762/bjoc.12.260 (PMC5238558; doi:10.3762/bjoc.12.260)
Supplement: File 1 — Experimental and copies of spectra. [file Beilstein_J_Org_Chem-12-2636-s001.pdf]

Supporting Information

for

Copper-catalyzed asymmetric sp^3 C–H arylation of tetrahydroisoquinoline mediated by a visible light photoredox catalyst

Pierre Querard[1], Inna Perepichka[1], Eli Zysman-Colman[2] and Chao-Jun Li*[1]

Address: [1]Department of Chemistry, FQRNT Center for Green Chemistry and Catalysis, McGill University, 801 Sherbrooke Street West, Montreal, Quebec H3A 0B8, Canada and [2]Organic Semiconductor Centre, EaStCHEM School of Chemistry, University of St Andrews, St Andrews, Fife, KY16 9ST, UK

Email: Prof. Chao-Jun Li - cj.li@mcgill.ca

*Corresponding author

Experimental and copies of spectra

Contents

**1.**	General information	S3
**2.**	General procedure for the synthesis of 2-phenyl-1,2,3,4-tetrahydroisoquinoline	S3
**3.**	Characterization of starting materials	S4
**4.**	Synthesis of $[Ir(ppy)_2(dtbbpy)](PF_6)$	S5
**5.**	Characterization data and HPLC chromatogram for the racemic and chiral tetrahydraisoquinoline derivatives	S6
**6.**	Effect of conditions on the coupling of 2-phenyl-1,2,3,4-tetrahydroisoquinoline with phenylboronic acid	S20
**7.**	NMR data for 1,2-diaryl-1,2,3,4-tetrahydraisoquinoline derivatives	S23

1. General information

Solvents and reagents were purchased from Sigma-Aldrich chemical company and were used without further purification unless otherwise specified. The glassware was oven-dried for at least 1 h prior to use. All reagents were weighed and handled in air, and backfilled under an inert atmosphere of nitrogen at room temperature. 2-Phenyltetrahydroisoquinolines **1** (Scheme 3)[1,2,3] and $[Ir(ppy)_2(dtbbpy)]PF_6$[4,5] were synthesized according to the published procedures and spectroscopic data were consistent (see text later) with those previously reported for these compounds. ^{1}H NMR and ^{13}C NMR spectra were obtained using Varian MERCURY- 400, and 500 MHz and a Bruker AV500 spectrometers operating respectively at 500 MHz and 126 MHz for ^{1}H and ^{13}C acquisitions. GC–MS and HRMS (ESI) spectra were conducted at the Mass Spectroscopy Facility at the Department of Chemistry, McGill University on a Thermo PolarisQ GC System and a JEOL JMS-700 instrument, respectively. The enantiomeric excess was determined by HPLC with an Agilent 1260 Infinity Diode Array detector (G 4212B) by using a Chiralcel OD-H column (25 cm × 4.6 mm, particle size 5 μm) and 4:96 isopropanol/hexane as an eluent (flow rate 0.4 mL/min, UV detection at 254 nm). Analytical thin-layer chromatography (TLC) was performed on Merck silica gel aluminium plates with F-254 indicator, visualised by irradiation with UV light.

2. General procedure for the synthesis of 2-phenyltetrahydroisoquinoline (1)[1]

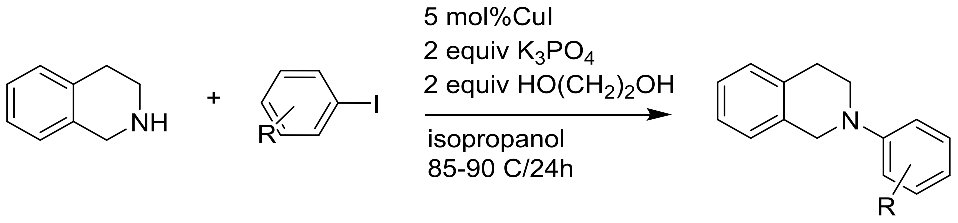

Copper(I) iodide (200 mg, 1.0 mmol) and potassium phosphate (4.25 g, 20.0 mmol) were placed into a 50 mL three-neck flask. The flask was evacuated and back-filled with argon. 2-Propanol (10.0 mL), ethylene glycol (1.11 mL), 1,2,3,4-tetrahydroisoquinoline (2.0 mL, 15 mmol) and iodobenzene (1.12 mL, 10.0 mmol) were added successively by syringe at room temperature. The reaction mixture was heated at 90 °C for 24 h and then allowed to cool to room temperature.

Diethyl ether (20 mL) and water (20 mL) were then added to the reaction mixture. The organic layer was extracted with diethyl ether (2 × 20 mL). The combined organic phases were washed with brine and dried over sodium sulfate. The solvent was removed and the residue was purified by column chromatography on silica gel using hexane/ethyl acetate (20:1) as an eluent.

3. Characterization of starting materials:

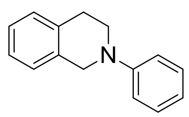

2-Phenyl-1,2,3,4-tetrahydroisoquinoline,[1] ^{1}H NMR (400 MHz, $CDCl_3$): δ 7.32-7.26 (m, 2 H), 7.21-7.15 (m, 4 H), 7.00 (d, *J* = 8.0 Hz, 2 H), 6.84 (t, *J* =8.0 Hz, 1 H), 4.42 (s, 2 H), 3.58 (t, *J* = 6 Hz, 2 H), 3.00 (t, *J*= 5.6 Hz, 2H).

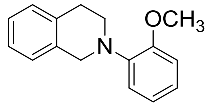

2-(2-Methoxyphenyl)-1,2,3,4-tetrahydroisoquinoline,[1] ^{1}H NMR (300 MHz, $CDCl_3$): δ 7.18-7.11 (m, 4 H), 7.03-7.0 (m, 2 H), 6.94-6.89 (m, 2 H), 4.31 (s, H), 3.89 (s, 3 H), 3.43 (t, *J* =6.0 Hz, 2 H), 2.99 (t, *J* = 6.0 Hz, 2H).

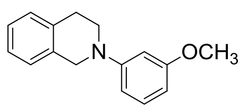

2-(3-Methoxyphenyl)-1,2,3,4-tetrahydroisoquinoline, [6] ^{1}H NMR (400 MHz, $CDCl_3$): δ 7.25–7.15 (m, 4 H), 6.62–6.59 (m, 1 H), 6.53–6.52 (m, 1H), 6.41-6.39 (m, 1 H), 4.42 (s, 2 H), 3.81 (s, 3 H), 3.56 (t, *J* =6.4 Hz, 2 H), 2.99 (t, *J* = 5.6 Hz, 2H).

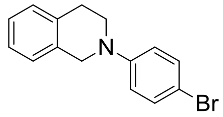

2-(4-Bromophenyl)-1,2,3,4-tetrahydroisoquinoline,[2] ^{1}H NMR (300 MHz,$CDCl_3$): δ 7.37-7.34 (d, *J* = 9.0 Hz, 2 H), 7.20-7.15 (m, 4 H), 6.84 (d, *J* = 9.0 Hz, 2 H), 4.38 (s, 2 H), 3.54 (t, *J* = 6.9 Hz, 2 H), 3.00 (t, *J* = 6.0 Hz, 2H).

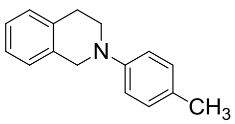

2-(4-Methylphenyl)-1,2,3,4-tetrahydroisoquinoline (1k),[2] ^{1}H NMR

(500MHz, $CDCl_3$): δ 7.18-7.11 (m, 4 H), 7.00 (d, *J* = 5 Hz, 2 H), 6.93 (d, *J* = 5 Hz, 2 H), 4.37 (s, 2 H), 3.52 (t, *J* = 5 Hz, 2 H), 2.99 (t, *J* = 5.5 Hz, 2 H), 2.29 (s, 1H).

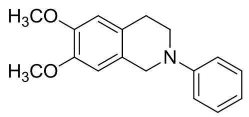

6,7-Dimethoxy-2-phenyl-1,2,3,4-tetrahydroisoquinoline,[7]
^{1}H NMR (300 MHz, $CDCl_3$): δ 7.33-7.25 (m, 2 H), 7.02-6.95 (m, 2 H), 6.87-6.78 (m, 1 H), 6.66, 6.65 (s, 2H), 4.34 (s, 2H), 3.88 (s, 3 H), 3.87 (s, 3H), 3.55 (t, *J* =5.8 Hz, 2 H), 2.90 (t, *J* = 5.8 Hz, 2H).

4. Synthesis of [Ir(ppy)$_2$(d*t*bbpy)](PF$_6$) [5,6]

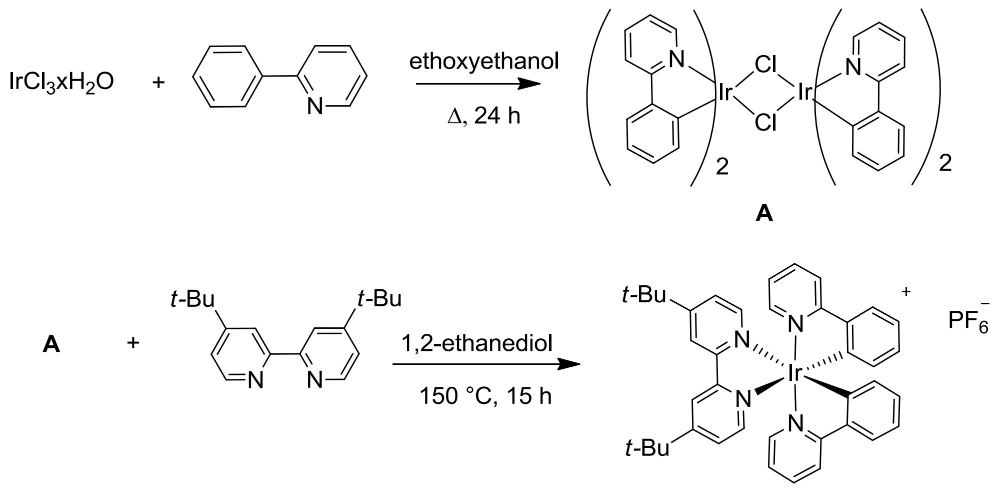

Scheme 1S: **Synthesis of [Ir(ppy)$_2$(d*t*bbpy)](PF$_6$)**.

Tetrakis(2-phenylpyridine-C2,N') (μ-dichloro)diiridium, (A, Scheme 1S) Iridium trichloride hydrate (0.389 g, 1.31 mmol) was combined with 2- phenylpyridine (0.76 g, 4.9 mmol), dissolved in a mixture of 2-ethoxyethanol (30 mL) and water (10 mL), and refluxed for 24 h. The solution was cooled to room temperature, and the yellow precipitate was collected on a glass filter frit. The precipitate was washed with ethanol (60 mL) and acetone (60 mL) and then dissolved in dichloromethane (75 mL) and filtered. Toluene (25 mL) and hexanes (10 mL) were added to the

filtrate, which was then reduced in volume by evaporation to 50 mL, and cooled to give crystals of $[Ir(ppy)_2Cl]_2$ (0.44 g, 75%) (**A**, Scheme 1S).

Synthesis of $[Ir(ppy)_2(dtbbpy)](PF_6)$ was adapted from literature procedures[6] for the analogous unsubstituted complex. A stirred suspension of 4,4′-di-*tert*-butyl-2,2′-dipyridyl (0.44 g, 0.88 mmol) and tetrakis(2-phenylpyridine-C,N)(μ-dichloro)diiridium, **A** (0.428g, 0.400 mmol) in 20 mL of 1,2-ethanediol under nitrogen was heated to 150 °C for 15 h. All the solids dissolved to yield a clear, yellow solution. After cooling the mixture to room temperature, 200 mL of water were added. The excess of the bipyridine ligand was removed through three extractions with diethyl ether (3 × 50 mL), and the aqueous layer was subsequently heated to 70 °C. NH_4PF_6 (2 g) in 20 mL of water was added, and the PF_6 salt of the iridium complex immediately precipitated. After cooling the suspension to 5 °C, the yellow solid was separated through filtration, dried, and recrystallized through acetonitrile/ether. Yield: 0.50 g (66%). ^{1}H NMR (acetone-d_6, 400 MHz): δ 8.88 (d, *J* =2.0 Hz, dtb-bpy-H3, 2H), 8.24 (ppy-H6, pyridine, 2H, d, *J* = 8), 7.99-7.93 (m, dtb-bpy-H6, 2H, ppy-H5, pyridine, 2H), 7.90 (ppy-H3, phenyl, 2H, dd, *J* = 7.2, 0.8 Hz), 7.79 (ppy-H6, phenyl, 2H, d, *J* = 6 Hz), 7.71 (dtb-bpy-H5, 2H, dd, *J* = 6.0, 2.0 Hz), 7.14 (ppy-H4, pyridine, 2H, dt, *J* = 7.2, 1.6 Hz), 7.04 (ppy-H4, phenyl, 2H, dt, *J*= 7.6, 0.8 Hz), 6.91 (ppy-H5, phenyl, 2H, dt, *J* = 6.8, 1.2 Hz), 6.34 (ppy-H3, pyridine, 2H, d, *J* = 8), 1.42 (18H, s).
HRMS (ESI) *m/z* calculated for $C_{40}H_{40}N_4Ir^+$ ($[M - PF_6]^+$) 769.2876, found 769.2866.

5. Characterization data and HPLC chromatogram for the racemic and chiral tetrahydraisoquinoline derivatives.

1,2-Diphenyl-1,2,3,4-tetrahydroisoquinoline (3a)

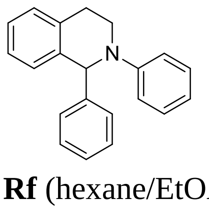

Use the general procedure described in the manuscript, compound **3a** was obtained from 2-phenyl-1,2,3,4-tetrahydroisoquinoline (0.1 mmol, 21.0 mg) and phenylboronic acid (0.3 mmol, 36.5 mg) as a white solid (21.4 mg) in 85% yield. **Rf** (hexane/EtOAc 3:0.5): 0.8.

^{1}H NMR ($CDCl_3$, 500 MHz): δ = 7.38 – 7.10 (m, 11H), 6.85 (m, 2H), 6.75 (m, 1H), 5.83 (s, 1H), 3.73 (dt, J = 11.1, 5.2 Hz, 1H), 3.52 (ddd, J = 11.1, 8.6, 5.2 Hz, 1H), 3.21 – 2.42 (m, 2H). **^{13}C NMR**

($CDCl_3$, 126 MHz): δ = 149.5, 143.0, 137.8, 135.7, 129.1, 128.2, 128.0, 127.7, 127.2, 127.0, 126.7, 126.1, 117.4, 113.8, 62.7, 43.8, 28.0. **HRMS (ESI) m/z:** $[M + H]^+$ calculated for $C_{21}H_{20}N$ 286.15903, found 286.15840.

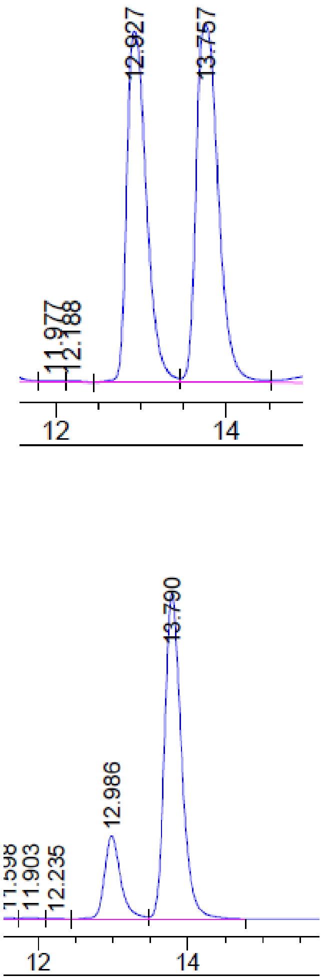

Peak	Retention Time / min	Area / %
Major Enantiomer	12.986	19.2879
Minor Enantiomer	13.790	78.4008

Figure 1S: HPLC Chromatogram for the two enantiomers of compound **3a** synthesized according to the procedure described in Table 4, entry 1.

2-(2-Methoxyphenyl)-1-phenyl-1,2,3,4-tetrahydroisoquinoline (3b)

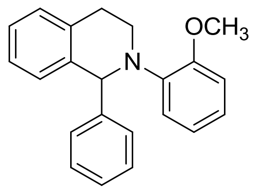

Use the general procedure described in the manuscript, compound **3b** was obtained from 2-(2-methoxyphenyl)-1,2,3,4-tetrahydroisoquinoline (0.1 mmol, 23.9 mg) and phenylboronic acid (0.3 mmol, 36.5 mg) as a white solid (17.6 mg) in 56% yield. **Rf** (hexane/EtOAc 3:0.5): 0.7.

^{1}H NMR ($CDCl_3$, 500 MHz): δ = 7.20 (m, 2H), 7.16 – 7.08 (m, 4H), 6.96 (m, 2H), 6.87 (m, 3H), 6.73 (m, 1H), 6.58 (m, 1H), 5.91 (s, 1H), 3.91 (s, 3H), 3.41 (ddd, J = 12.2, 10.3, 4.5 Hz, 1H), 3.31 (ddd, J = 12.2, 6.2, 3.0 Hz, 1H), 3.14 (ddd, J = 16.4, 10.3, 6.2 Hz, 1H), 2.96 (dt, J = 16.4, 3.0 Hz, 1H). **^{13}C NMR ($CDCl_3$, 126 MHz):** δ = 153.0, 142.0, 140.0, 137.2, 135.0, 129.3, 128.8, 128.7, 127.3, 126.7, 126.2, 125.5, 122.9, 121.9, 120.8, 111.5, 62.7, 55.6, 43.0, 28.7. **HRMS (ESI) m/z:** $[M + H]^+$ calculated for $C_{22}H_{22}NO$ 316.16959, found 316.16980.

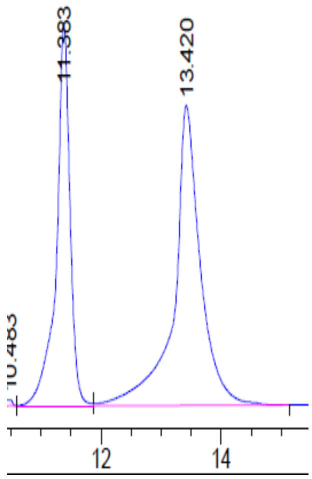

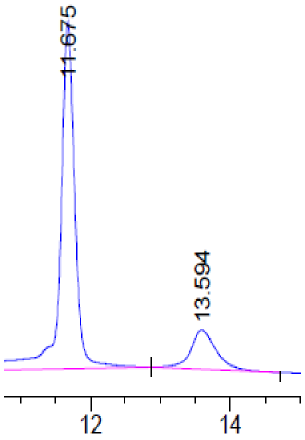

Peak	Retention Time / min	Area / %
Major Enantiomer	11.675	60.1237
Minor Enantiomer	13.594	11.9011

2-(3-Methoxyphenyl)-1-phenyl-1,2,3,4-tetrahydroisoquinoline (3c)

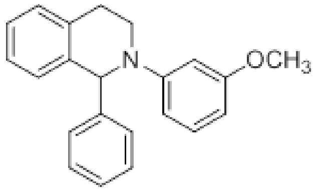

Use the general procedure in the manuscript, compound **3c** was obtained from 2-(3-methoxyphenyl)-1,2,3,4-tetrahydroisoquinoline (0.1 mmol, 23.9 mg) and phenylboronic acid (0.3 mmol, 36.5 mg) as a white solid (19.2 mg) in 61% yield. **Rf** (hexane/EtOAc 3:0.5): 0.7.

^{1}H NMR ($CDCl_3$, 500 MHz): δ = 7.37 – 6.99 (m, 10H), 6.60 – 6.14 (m, 3H), 5.83 (s, 1H), 3.76 (s, 3H), 3.75 – 3.67 (m, 1H), 3.51 (m, 1H), 2.93 (m, 2H). **^{13}C NMR ($CDCl_3$, 126 MHz):** δ = 160.6, 150.8, 143.0, 137.8, 135.7, 129.8, 128.2, 128.0, 127.7, 127.1, 127.0, 126.7, 126.1, 106.7, 102.0, 100.2, 62.7, 55.1, 43.9, 28.0.**HRMS (ESI) m/z:** $[M + H]^+$ calculated for $C_{22}H_{22}NO$ 316.16959, found 316.16950.

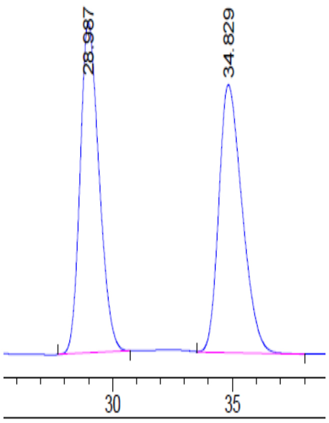

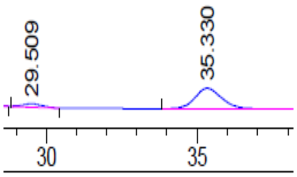

Peak	Retention Time / min	Area / %
Major Enantiomer	29.509	1.8996
Minor Enantiomer	35.330	16.4503

2-(4-Methoxyphenyl)-1-phenyl-1,2,3,4-tetrahydroisoquinoline (3d)

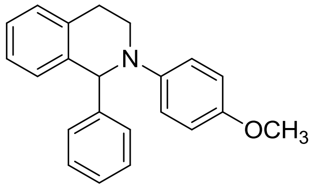

Use the general procedure described above, compound **3d** was obtained from 2-(4-methoxyphenyl)-1,2,3,4-tetrahydroisoquinoline (0.1 mmol, 23.9 mg) and phenylboronic acid (0.3 mmol, 36.5 mg) as a white solid (16.7 mg) in 53% yield. **Rf** (hexane/EtOAc 3:0.5): 0.7

^{1}H NMR ($CDCl_3$, 500 MHz): δ = 7.28 – 7.15 (m, 9H), 6.89 – 6.79 (m, 4H), 5.69 (s, 1H), 3.77 (s, 3H), 3.61 (ddd, J = 12.0, 6.7, 5.2 Hz, 1H), 3.44 (ddd, J = 12.0, 7.2, 5.2 Hz, 1H), 3.04 – 2.92 (m,

2H). **^{13}C NMR ($CDCl_3$, 126 MHz):** δ = 152.8, 144.4, 143.2, 137.6, 135.5, 128.3, 128.1, 128.09, 128.02, 126.8, 126.7, 125.9, 117.7, 114.4, 64.3, 55.6, 44.5, 28.1.

HRMS (ESI) m/z: $[M + H]^+$ calculated for $C_{22}H_{22}NO$ 316.16959, found 316.16847.

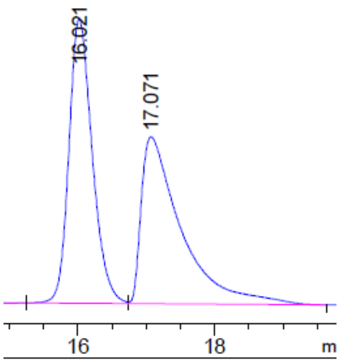

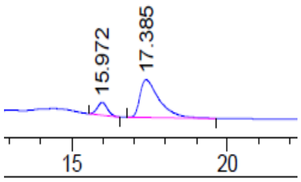

Peak	Retention Time / min	Area / %
Major Enantiomer	15.972	3.1868
Minor Enantiomer	17.385	18.4472

1-Phenyl-2-(*p*-tolyl)-1,2,3,4-tetrahydroisoquinoline (3[e])

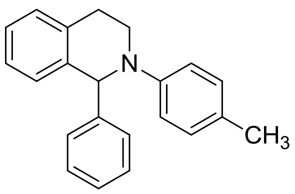

Use the general procedure described in the manuscript, compound **3e** was obtained from 2-(*p*-tolyl)-1,2,3,4-tetrahydroisoquinoline (0.1 mmol, 22.3 mg) and phenylboronic acid (0.3 mmol, 36.5 mg) as a white solid (23.3 mg) in 78% yield. **Rf** (hexane/EtOAc 3:0.5): 0.75.

^{1}H NMR ($CDCl_3$, 500 MHz): δ = 7.31 – 7.12 (m, 9H), 7.04 (m, 2H), 6.79 (m, 2H), 5.79 (s, 1H), 3.68 (dt, J = 11.4, 5.6 Hz, 1H), 3.49 (ddd, J = 11.4, 8.2, 5.6 Hz, 1H), 3.19 – 2.68 (m, 2H), 2.26 (s, 3H). **^{13}C NMR ($CDCl_3$, 126 MHz):** δ = 147.5, 143.3, 137.8, 135.7, 129.6, 128.7, 128.17, 128.13, 127.9, 127.5, 126.9, 126.7, 126.0, 114.5, 63.1, 43.9, 27.9, 20.3. **HRMS (ESI) m/z:** $[M + H]^+$ calculated for $C_{22}H_{24}NO$ 300.17468, found 300.17507.

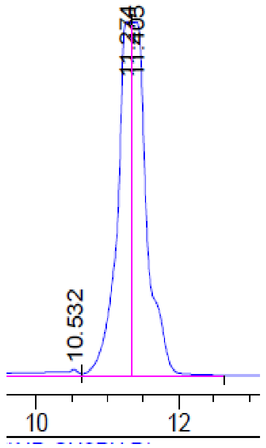

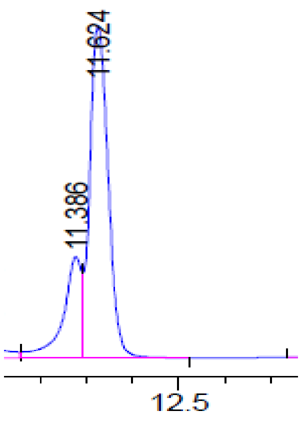

Peak	Retention Time / min	Area / %
Major Enantiomer	11.386	20.9969
Minor Enantiomer	11.624	68.4203

2-(4-Bromophenyl)-1-phenyl-1,2,3,4-tetrahydroisoquinoline (3f)

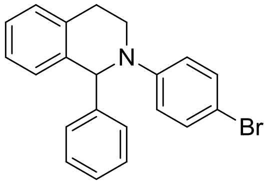

Use the general procedure described above, compound **3f** was obtained from 2-(4-bromophenyl)-1,2,3,4-tetrahydroisoquinoline (0.1 mmol, 28.8 mg) and phenylboronic acid (0.3 mmol, 36.5 mg) as a white solid (29.1 mg) in 80% yield. **Rf** (hexane/EtOAc 3:0.5): 0.85.

^{1}H NMR ($CDCl_3$, 500 MHz): δ = 7.35 – 7.09 (m, 11H), 6.71 (m, 2H), 5.76 (s, 1H), 3.71 (dt, J = 11.2, 5.3 Hz, 1H), 3.47 (dt, J = 11.2, 7.2 Hz, 1H), 2.94 (m, 2H). **^{13}C NMR ($CDCl_3$, 126 MHz):** δ = 148.3, 142.4, 137.5, 135.4, 131.8, 128.3, 128.0, 127.7, 127.2, 127.1, 126.9, 126.3, 115.4, 109.4, 62.8, 44.1, 27.9. **HRMS (ESI) m/z:** $[M + H]^+$ calculated for $C_{21}H_{19}NBr$ 364.06954, found 364.06927.

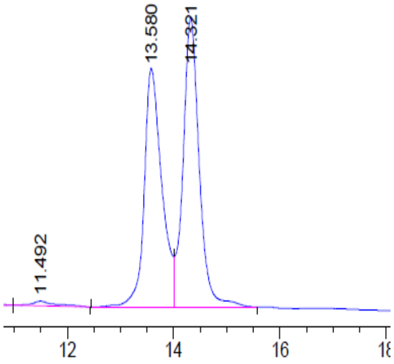

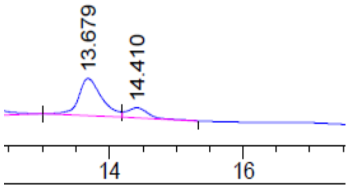

Peak	Retention Time / min	Area / %
Major Enantiomer	13.679	6.0486
Minor Enantiomer	14.410	1.4782

1-(4-(2-Phenyl-1,2,3,4-tetrahydroisoquinolin-1-yl)phenyl)ethanone (3g)

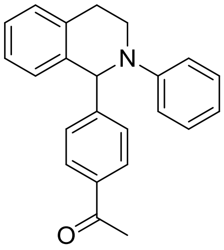

Use the general procedure described above, compound **3g** was obtained from 2-phenyl-1,2,3,4-tetrahydroisoquinoline (0.1 mmol, 21.0 mg) and (4-acetylphenyl)boronic acid (0.3 mmol, 49.2 mg) as a white solid (18.6 mg) in 57% yield. **Rf** (hexane/EtOAc 3:0.5): 0.45

^{1}H NMR ($CDCl_3$, 500 MHz): δ = 7.91 – 7.83 (m, 2H), 7.43 – 7.37 (m, 2H), 7.35 – 7.17 (m, 6H), 6.95 – 6.76 (m, 3H), 5.87 (s, 1H), 3.78 (dt, *J* = 10.9, 5.2 Hz, 1H), 3.61 – 3.50 (m, 1H), 3.01 (q, *J* = 7.5, 5.2 Hz, 1H), 2.57 (s, 2H). **^{13}C NMR ($CDCl_3$, 126 MHz):** δ = 197.7, 148.5, 137.0, 135.9, 135.6, 129.2, 128.4, 128.2, 127.7, 127.5, 127.4, 126.4, 124.4, 118.0, 114.0, 63.0, 44.2, 28.1, 26.6. **HRMS (ESI) m/z:** $[M + H]^+$ calculated for $C_{23}H_{22}NO$ 328.1701, found 328.1697.

1-(4-Fluorophenyl)-2-phenyl-1,2,3,4-tetrahydroisoquinoline (3h)

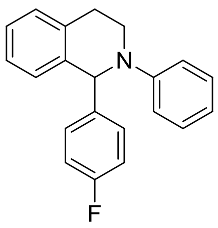

Use the general procedure described above, compound **3h** was obtained from 2-phenyl-1,2,3,4-tetrahydroisoquinoline (0.1 mmol, 21.0 mg) and (4-fluorophenyl)boronic acid (0.3 mmol, 42.0 mg) as a white solid (17.0 mg) in 56% yield. **Rf** (hexane/EtOAc 3:0.5): 0.55.

^{1}H NMR ($CDCl_3$, 500 MHz): δ = 7.27 – 7.15 (m, 8H), 6.95 – 6.89 (m, 2H), 6.87 – 6.83 (m, 2H), 6.79 – 6.70 (m, 1H), 5.80 (s, 1H), 3.68 (dt, J = 11.4, 5.2 Hz, 1H), 3.50 (ddd, J = 11.4, 8.7, 5.2 Hz, 1H), 2.97 (dt, J = 15.6, 5.2 Hz, 1H), 2.88 (ddd, J = 15.6, 8.7, 5.3 Hz, H). **^{13}C NMR ($CDCl_3$, 126 MHz):** δ = 162.7, 160.7, 149.4, 138.75, 138.73, 137.5, 135.6, 129.1, 128.9, 128.8, 128.2, 127.7, 127.1, 126.1, 117.8, 115.0, 114.8, 114.2, 104.9, 62.2, 43.6, 27.9. **^{19}F NMR ($CDCl_3$, 470 MHz):** δ = -104.5. **HRMS (ESI) m/z:** $[M + H]^+$ calculated for $C_{21}H_{19}NF$ 304.1496, found 304.1496.

1-(2,4-Difluorophenyl)-2-phenyl-1,2,3,4-tetrahydroisoquinoline (3i)

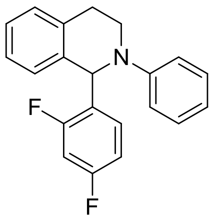

Use the general procedure described above, compound **3i** was obtained from 2-phenyl-1,2,3,4-tetrahydroisoquinoline (0.1 mmol, 21.0 mg) and (2,4-difluorophenyl)boronic acid (0.3 mmol, 47.3 mg) as a white solid (17.3 mg) in 54% yield. **Rf** (hexane/EtOAc 3:0.5): 0.5.

^{1}H NMR ($CDCl_3$, 500 MHz): δ = 7.29 – 7.17 (m, 6H), 7.17 – 7.12 (m, 1H), 6.94 – 6.88 (m, 2H), 6.84 – 6.77 (m, 2H), 6.77 – 6.71 (m, 1H), 6.11 (s, 1H), 3.78 (dt, J = 11.7, 6.0 Hz, 1H), 3.59 (dt, J = 11.7, 6.0 Hz, 1H), 3.07 (t, J = 6.0 Hz, 2H). **^{13}C NMR ($CDCl_3$, 126 MHz):** δ = 163.0, 162.9, 161.4, 161.3, 161.0, 160.9, 149.1, 136.4, 135.4, 130.2, 130.19, 130.16, 130.11, 129.1, 128.2, 127.96, 127.93, 127.29, 127.26, 127.18, 127.15, 127.0, 126.4, 118.5, 115.1, 111.04, 111.01, 110.87, 110.84, 104.1, 103.9, 103.7, 57.0, 44.0, 28.0. **^{19}F NMR ($CDCl_3$, 470 MHz):** δ = -111.5, -111.9. **HRMS (ESI) m/z:** $[M + H]^+$ calculated for $C_{21}H_{18}NF_2$ 322.1402, found 322.1394.

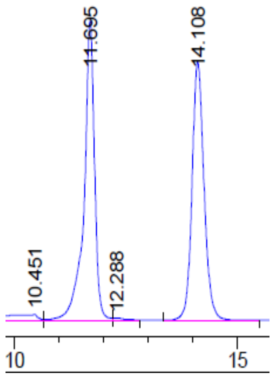

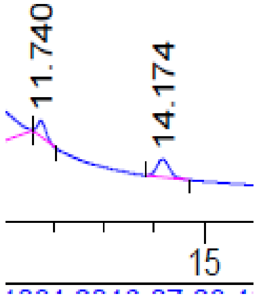

Peak	Retention Time / min	Area / %
Major Enantiomer	11.740	1.8420
Minor Enantiomer	14.174	3.0673

1-(3,5-Bis(trifluoromethyl)phenyl)-2-phenyl-1,2,3,4-tetrahydroisoquinoline (3j)

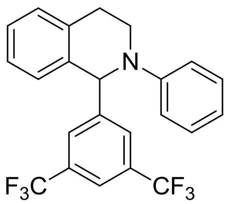

Use the general procedure described above, compound **3j** was obtained from 2-phenyl-1,2,3,4-tetrahydroisoquinoline (0.1 mmol, 21.0 mg) and (3,5-bis(trifluoromethyl)phenyl)boronic acid (0.3 mmol, 77.3 mg) as a white solid (13.9 mg) in 33% yield. **Rf** (hexane/EtOAc 3:0.5): 0.35.

^{1}H NMR ($CDCl_3$, 500 MHz): δ = 7.72 (s, 1H), 7.69 (s, 2H), 7.29 – 7.20 (m, 6H), 7.02 – 6.72 (m, 3H), 5.85 (s, 1H), 3.65 (ddd, J = 11.5, 6.2, 5.0 Hz, 1H), 3.51 (ddd, J = 11.5, 8.5, 4.8 Hz, 1H), 3.01 (ddd, J = 15.8, 6.2, 4.8 Hz, 1H), 2.86 (ddd, J = 15.7, 8.5, 5.0 Hz, 1H). **^{13}C NMR ($CDCl_3$, 126 MHz):** δ = 149.1, 146.3, 135.7, 135.6, 131.8, 131.6, 131.3, 131.0, 129.3, 128.6, 127.79, 127.70, 127.4, 126.6, 124.3, 122.1, 121.1, 119.0, 114.9, 104.9, 63.0, 44.0, 27.8. **^{19}F NMR ($CDCl_3$, 470**

MHz): δ = -62.7. **HRMS (ESI) m/z:** $[M + H]^+$ calculated for $C_{23}H_{18}NF_6$ 422.1338, found 422.1332.

1-(4-(tert-Butyl)phenyl)-2-phenyl-1,2,3,4-tetrahydroisoquinoline (3k)

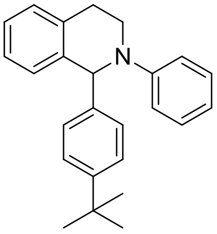

Use the general procedure described above, compound **3k** was obtained from 2-phenyl-1,2,3,4-tetrahydroisoquinoline (0.1 mmol, 21.0 mg) and (4-(tert-butyl)phenyl)boronic acid (0.3 mmol, 53.4 mg) as a white solid (20.1 mg) in 59% yield. **Rf** (hexane/EtOAc 3:0.5): 0.9.

^{1}H NMR ($CDCl_3$, 500 MHz): δ = 7.37 – 7.06 (m, 10H), 6.87 (m, 2H), 6.79 – 6.66 (m, 1H), 5.83 (s, 1H), 3.74 – 3.71 (m, 1H), 3.59 – 3.45 (m, 1H), 3.01 – 2.81 (m, 2H), 1.27 (s, 9H). **^{13}C NMR ($CDCl_3$, 126 MHz):** δ = 149.5, 139.9, 138.0, 135.7, 131.7, 129.1, 128.0, 127.8, 126.9, 126.8, 126.6, 126.0, 125.2, 125.1, 117.1, 115.0, 113.5, 62.3, 43.6, 34.3, 31.3, 27.8. **HRMS (ESI) m/z:** $[M + H]^+$ calculated for $C_{25}H_{28}N$ 342.2216, found 342.2211.

1-(3-Methoxyphenyl)-2-phenyl-1,2,3,4-tetrahydroisoquinoline (3l)

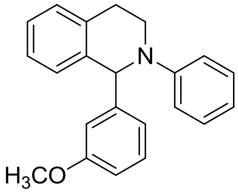

Use the general procedure described above, compound **3l** was obtained from 2-phenyl-1,2,3,4-tetrahydroisoquinoline (0.1 mmol, 21.0 mg) and (3-methoxyphenyl)boronic acid (0.3 mmol, 45.6 mg) as a white solid (23.3 mg) in 74% yield. **Rf** (hexane/EtOAc 3:0.5): 0.55

^{1}H NMR ($CDCl_3$, 500 MHz): δ = 7.36 – 7.31 (m, 1H), 7.30 – 7.18 (m, 6H), 6.94 – 6.85 (m, 4H), 6.83 – 6.74 (m, 2H), 5.84 (s, 1H), 3.91 – 3.75 (m, 4H), 3.59 – 3.51 (m, 1H), 2.99 (m, 2H). **^{13}C NMR ($CDCl_3$, 126 MHz):** δ = 159.5, 149.5, 145.0, 137.7, 135.7, 129.2, 129.1, 128.1, 127.8, 127.1, 126.2, 119.7, 117.5, 113.9, 113.5, 111.6, 62.9, 55.1, 43.8, 28.0. **HRMS (ESI) m/z:** $[M + H]^+$ calculated for $C_{22}H_{22}NO$ 316.1701, found 316.1696.

2-Phenyl-1-(4-vinylphenyl)-1,2,3,4-tetrahydroisoquinoline (3m)

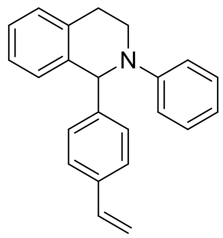

Use the general procedure described above, compound **3m** was obtained from 2-phenyl-1,2,3,4-tetrahydroisoquinoline (0.1 mmol, 21.0 mg) and (4-vinylphenyl) boronic acid (0.3 mmol, 44.4 mg) as a white solid (18.7 mg) in 60% yield. **Rf** (hexane/EtOAc 3:0.5): 0.9

^{1}H NMR ($CDCl_3$, 500 MHz): δ = 7.34 – 7.30 (m, 3H), 7.29 – 7.27 (m, 1H), 7.27 – 7.23 (m, 5H), 7.22 – 7.18 (m, 1H), 6.91 – 6.86 (m, 1H), 6.82 – 6.76 (m, 1H), 6.69 (dd, J = 17.6, 10.9 Hz, 1H), 5.85 (s, 1H), 5.71 (dd, J = 17.6, 0.9 Hz, 1H), 5.22 (dd, J = 10.9, 0.9 Hz, 1H), 3.76 (dt, J = 11.0, 5.1 Hz, 0H), 3.54 (ddd, J = 11.0, 8.7, 5.1 Hz, 1H), 3.03 – 2.92 (m, 1H). **^{13}C NMR ($CDCl_3$, 126 MHz):** δ = 149.5, 142.8, 137.7, 136.4, 136.2, 135.7, 129.1, 128.1, 127.7, 127.4, 127.0, 126.2, 126.1, 117.5, 113.9, 113.6, 62.6, 43.8, 28.1. **HRMS (ESI) m/z:** $[M + H]^+$ calculated for $C_{23}H_{22}N$ 312.1752, found 312.1752.

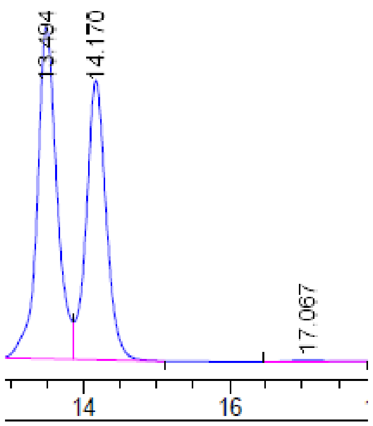

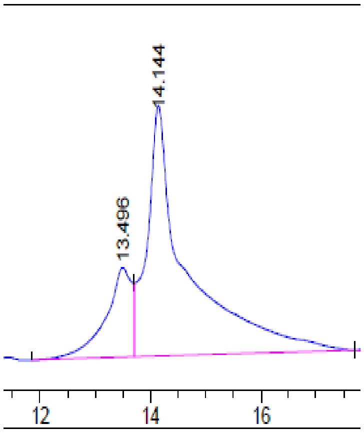

Peak	Retention Time / min	Area / %
Major Enantiomer	13.496	10.2151
Minor Enantiomer	14.144	43.9473

6,7-Dimethoxy-1-(3-methoxyphenyl)-2-phenyl-1,2,3,4-tetrahydroisoquinoline (3n)

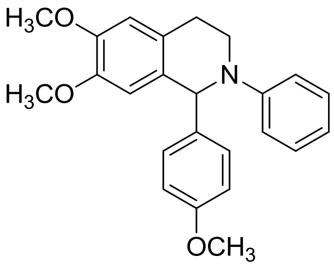

Use the general procedure described above, compound **3n** was obtained from 6,7-dimethoxy-2-phenyl-1,2,3,4-tetrahydroisoquinoline (0.1 mmol, 26.9 mg) and (4-methoxyphenyl)boronic acid (0.3 mmol, 45.6 mg) as a white solid (27.4 mg) in 73% yield. **Rf** (hexane/EtOAc 3:0.5): 0.2

^{1}H NMR ($CDCl_3$, 500 MHz): δ = 7.29 – 7.22 (m, 2H), 7.17 – 7.08 (m, 2H), 6.95 – 6.92 (m, 2H), 6.86 – 6.77 (m, 3H), 6.74 – 6.71 (m, 1H), 6.70 – 6.68 (m, 1H), 5.76 (s, 1H), 3.89 (s, 3H), 3.86 (s, 3H), 3.78 (s, 3H), 3.65 – 3.48 (m, 2H), 3.01 – 2.88 (m, 1H), 2.80 – 2.77 (m, 1H). **^{13}C NMR ($CDCl_3$, 126 MHz):** δ = 158.5, 149.8, 147.8, 147.2, 135.4, 129.1, 128.8, 127.6, 117.9, 115.0, 114.7, 113.4, 111.2, 111.0, 61.9, 56.0, 55.9, 55.2, 42.91, 27.0. **HRMS (ESI) m/z:** $[M + H]^+$ calculated for $C_{24}H_{25}NO_3$ 376.1907, found 376.1914.

6. **Effect of conditions on the coupling of 2-phenyl-1,2,3,4-tetrahydroisoquinoline with phenylboronic acid**

Table S1: Effect of conditions on the coupling of 2-phenyl-tetrahydroisoquinoline with phenylboronic acid.

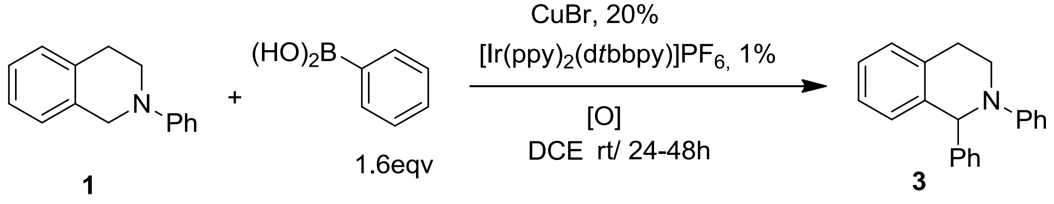

Entry[a]	Oxidant	**1**,%	**3**, Yield,%
1	T-HYDRO	ND[b]	14
2	TBHP	29	19
3	TBP	50	ND[b]
4	$(BzO)_2$	ND[b]	ND[b]
5	DDQ	ND[b]	ND[b]
6	dicumyl peroxide	40	ND[b]
7	*tert*-butyl peroxybenzoate	ND[b]	ND[b]
8	*tert*-butyl peracetate	18	trace

[a] Conditions: 0.1 mmol 2-phenyltetrahydroisoquinoline, 0.16 mmol phenylboronic acid, 1 mol % $[Ir(ppy)_2(d\mathit{t}bbpy)]PF_6$, 20 mol % copper salt, and 0.2 mmol [O]. The yield was calculated by ^1H NMR integration method using CH_2Br_2 as an internal standard. [b] The desired product was not detected.

Table S2: Effect of conditions on the coupling of 2-phenyl-tetrahydroisoquinoline with phenylboronic acid.

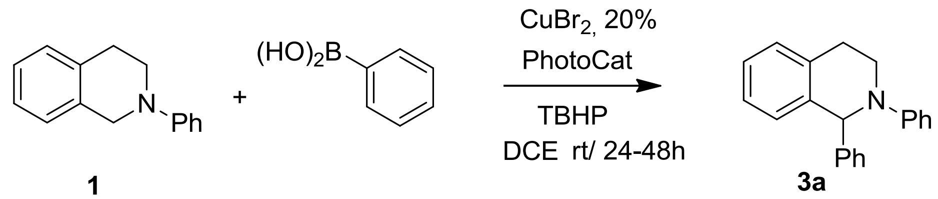

entry	photo catalyst using	**3a**, % NMR
1	$[Ir(ppy)_2(dtbbpy)]PF_6$	85
2	$[Ir(dF(CF_3)ppy)_2(dtbbpy)]PF_6$	29
3	$[Ru(bpy)_3](PF_6)_2$	72

[a] Conditions: 0.1 mmol 2-phenyl-tetrahydroisoquinoline, 0.3 mmol phenylboronic acid, 1 mol % $[Ir(ppy)_2(dtbbpy)PF_6$, 20 mol % copper salt, and 0.2 mmol TBHP. The yield was calculated by ^{1}H NMR integration method using CH_2Br_2 as an internal standard.

Table S3: Effect of chiral ligand on the enantioselectivity of coupling 2-phenyl-tetrahydroisoquinoline with phenylboronic acid at 4 C.[a]

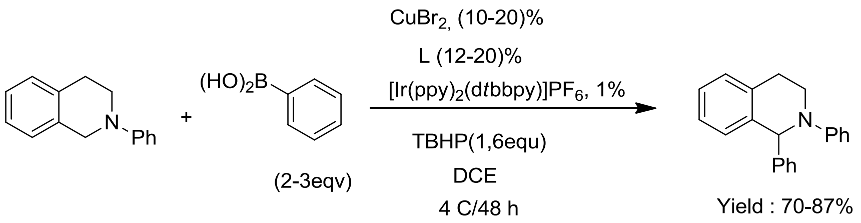

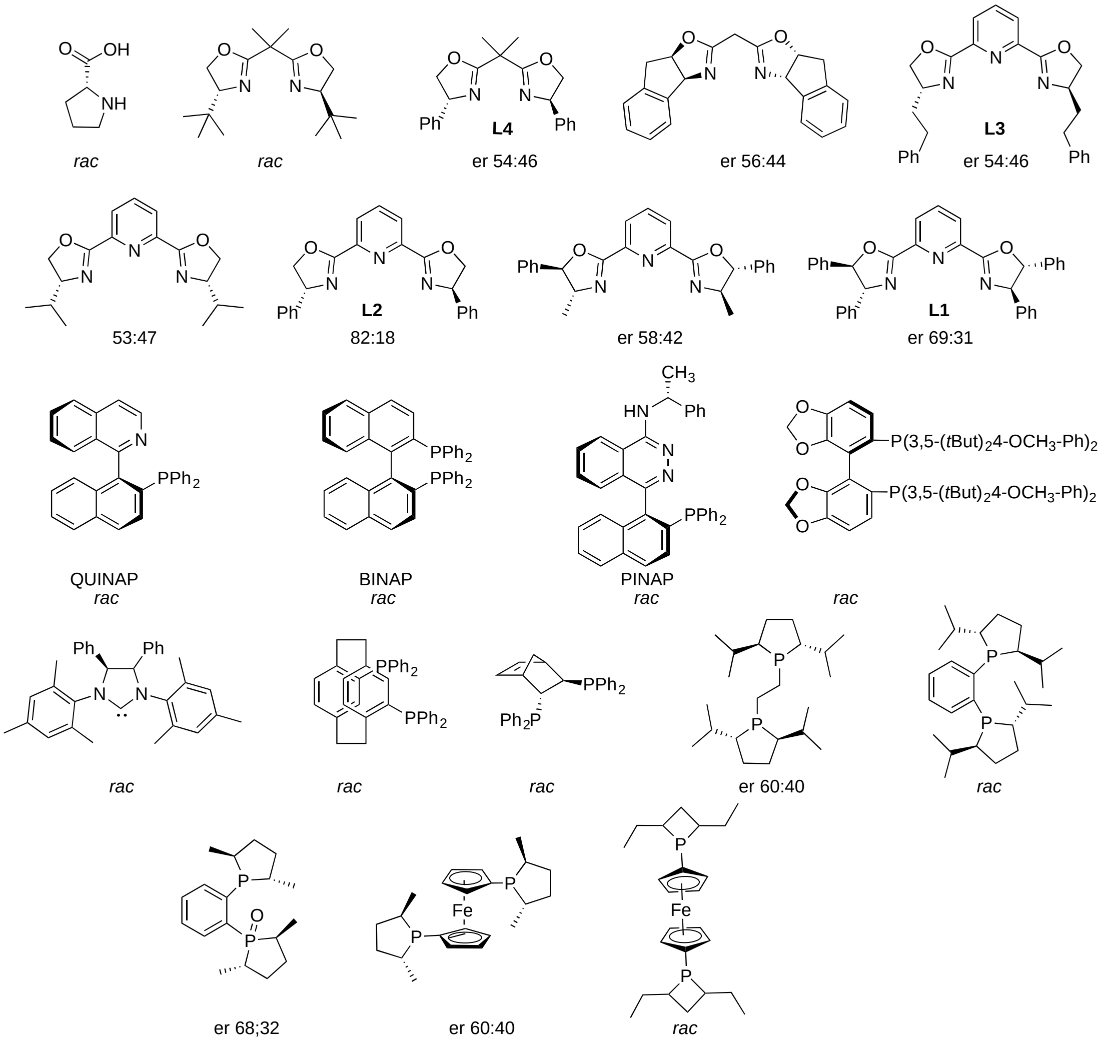

[a]Reaction conditions: THIQs (0.10 mmol), arylboronic acid (0.30 mmol), TBHP (0.2 mmol), $[Ir(ppy)_2(dtbbpy)]PF_6$ (0.001 mmol), CuBr (0.01 mmol), **L*** (0.012 mmol), DCE (0.5 mL), under argon atmosphere. All reported yields enantiomeric ratios were determined using a Chiralcel OD-H column and 96:4 hexane/isopropanol as an eluent.

7. NMR data for 1-aryl-2-aryl-1,2,3,4-tetrahydroisoquinoline derivatives

3a

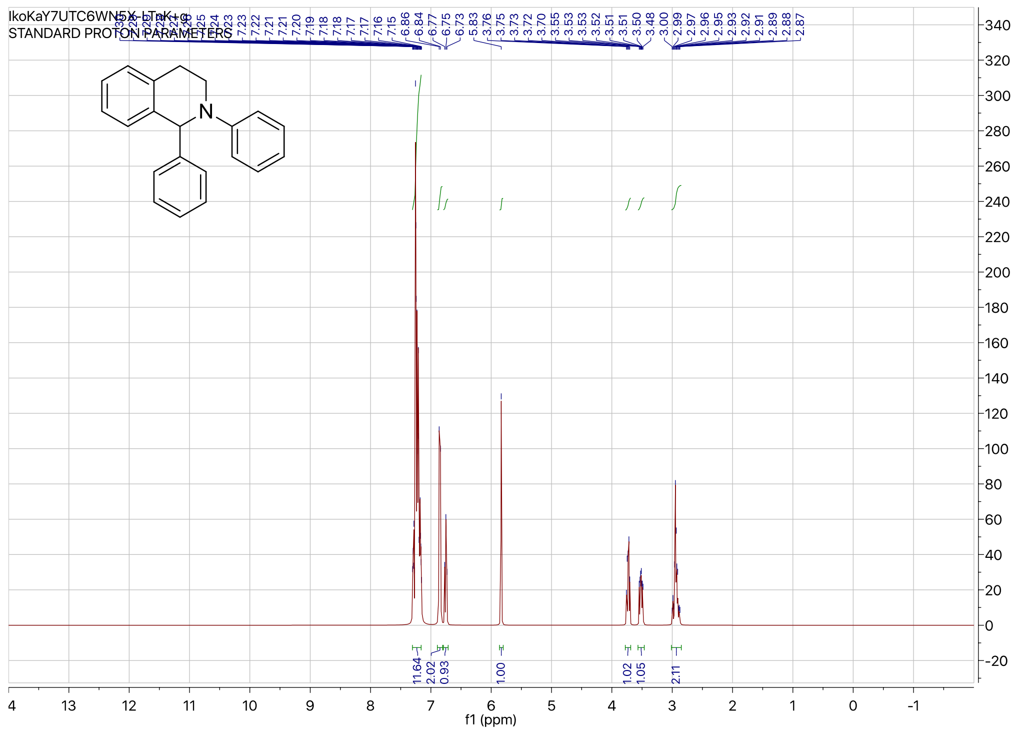

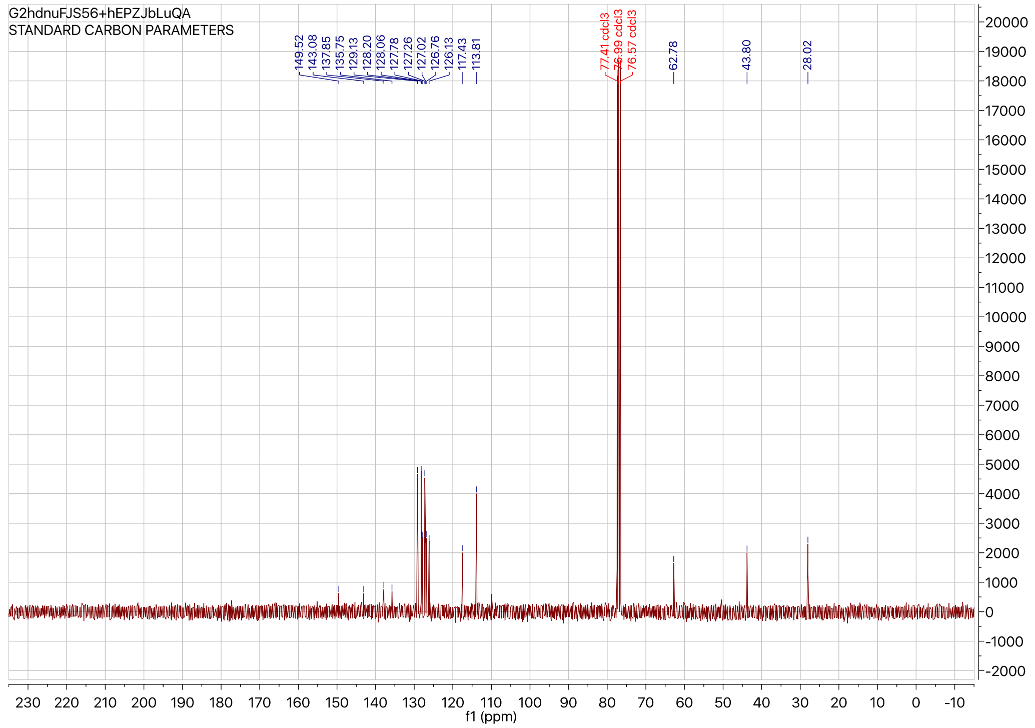

3b

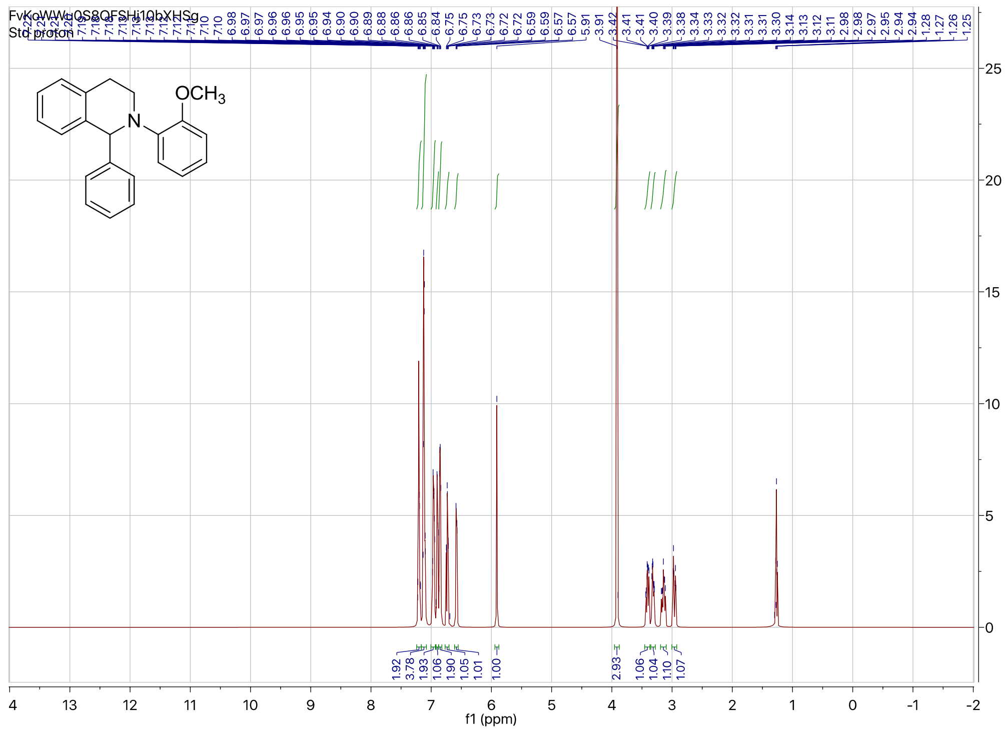
FvKoWWu0S8QFSHj10bXHSg
Std proton
OCH3
N
1.92
3.78
1.93
1.06
1.90
1.05
1.01
1.00
2.93
1.06
1.04
1.10
1.07
f1 (ppm)

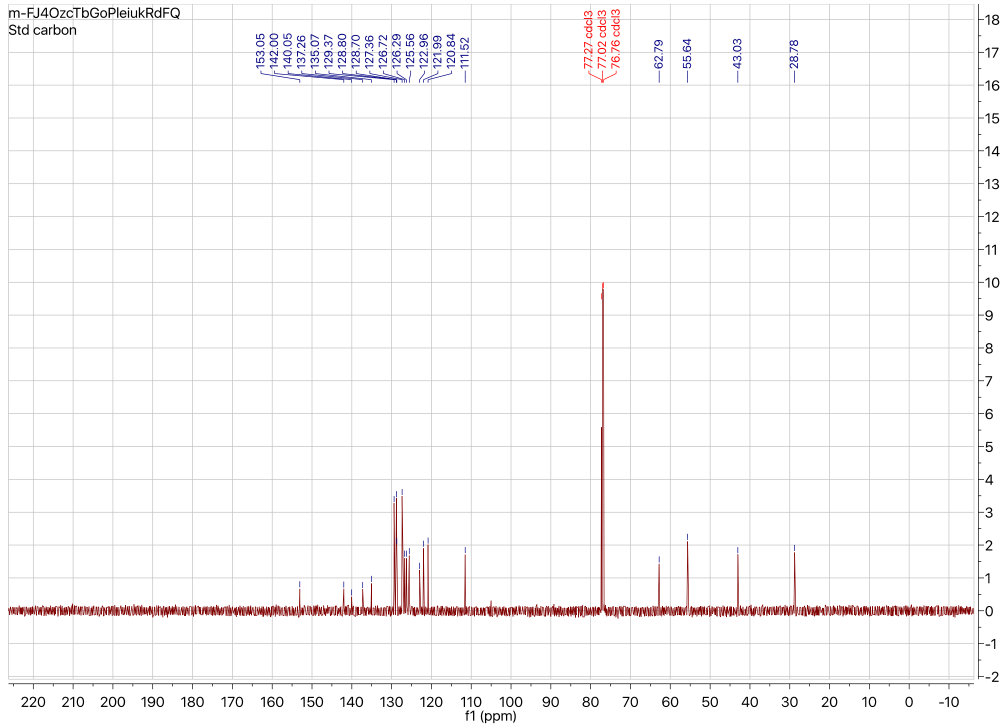
m-FJ4OzcTbGoPleiukRdFQ
Std carbon
153.05
142.00
140.05
137.26
135.07
129.37
128.80
128.70
127.36
126.72
126.29
125.56
122.96
121.99
120.84
111.52
77.27 cdcl3
77.02 cdcl3
76.76 cdcl3
62.79
55.64
43.03
28.78
f1 (ppm)

3c

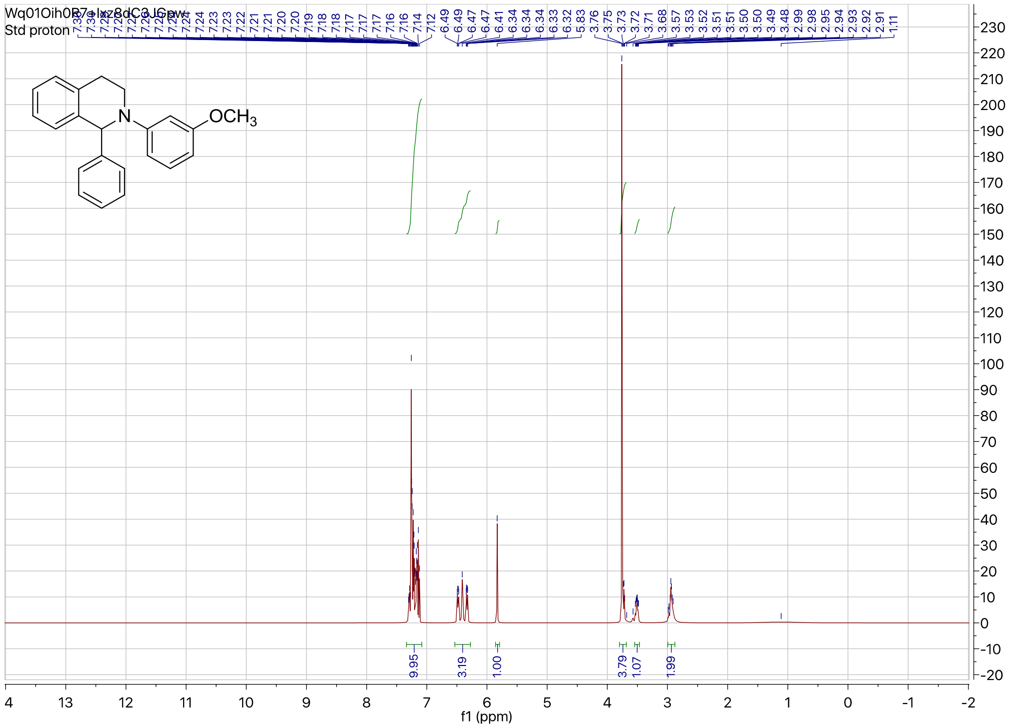
Std proton
OCH3
N
f1 (ppm)
9.95
3.19
1.00
3.79
1.07
1.99

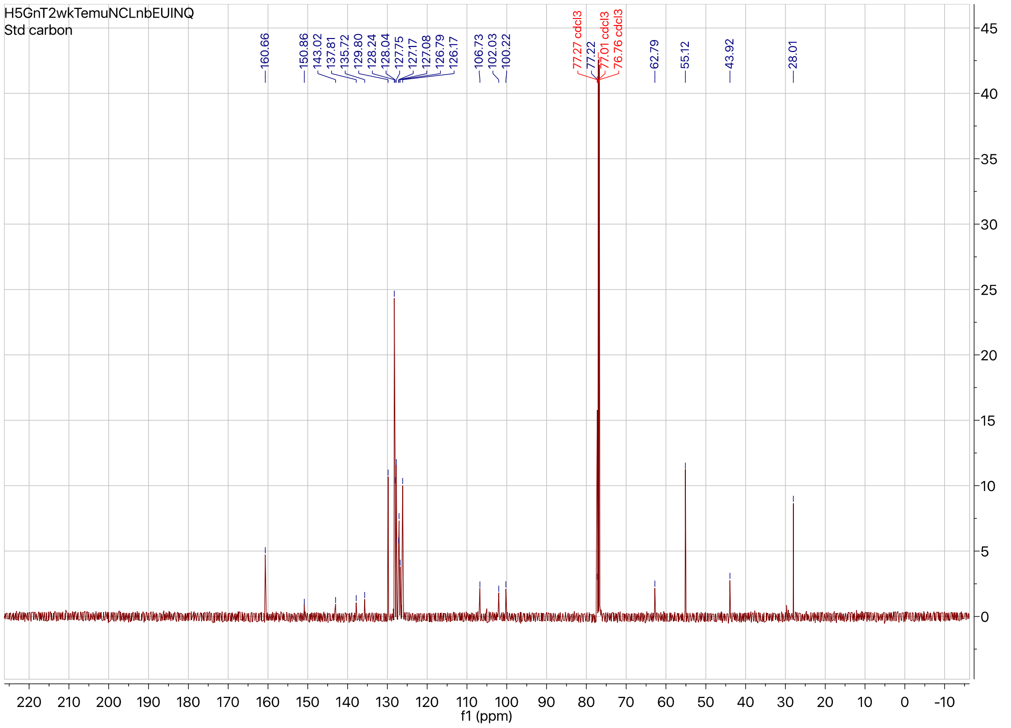
H5GnT2wkTemuNCLnbEUlNQ
Std carbon
160.66
150.86
143.02
137.81
135.72
129.80
128.24
128.04
127.75
127.17
127.08
126.79
126.17
106.73
102.03
100.22
77.27 cdcl3
77.22
77.01 cdcl3
76.76 cdcl3
62.79
55.12
43.92
28.01
f1 (ppm)

3d

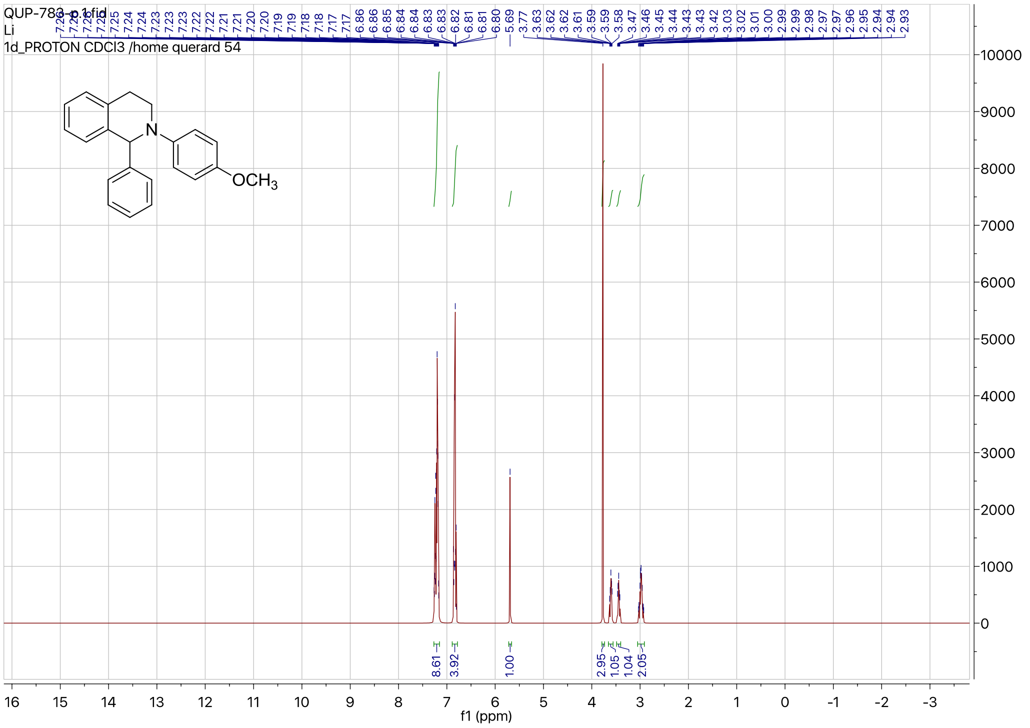

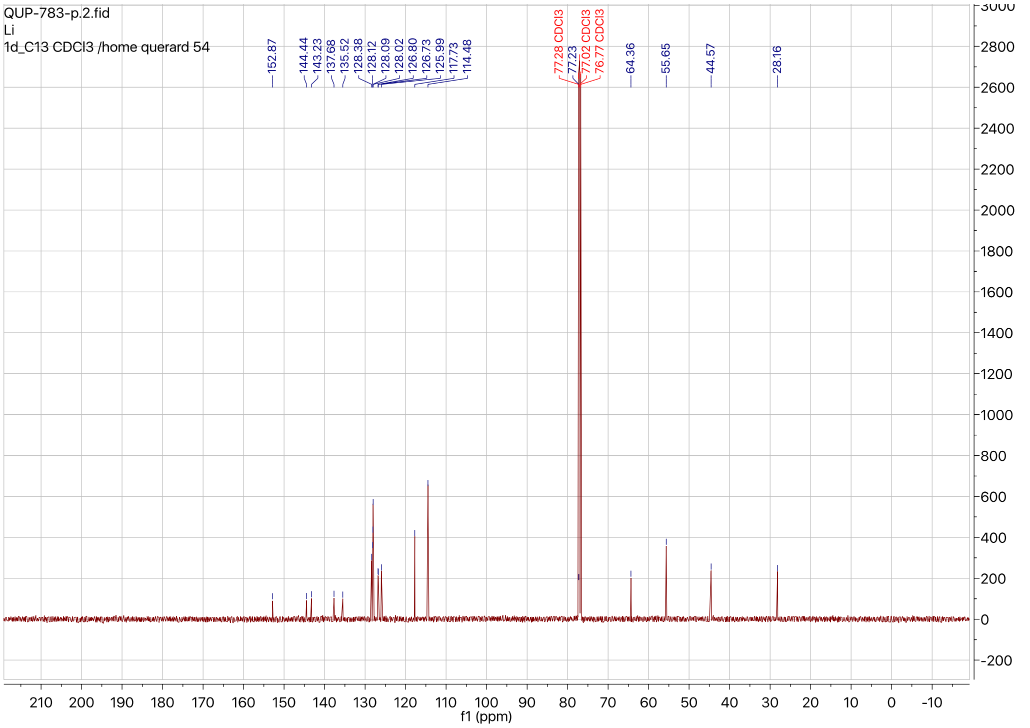

3e

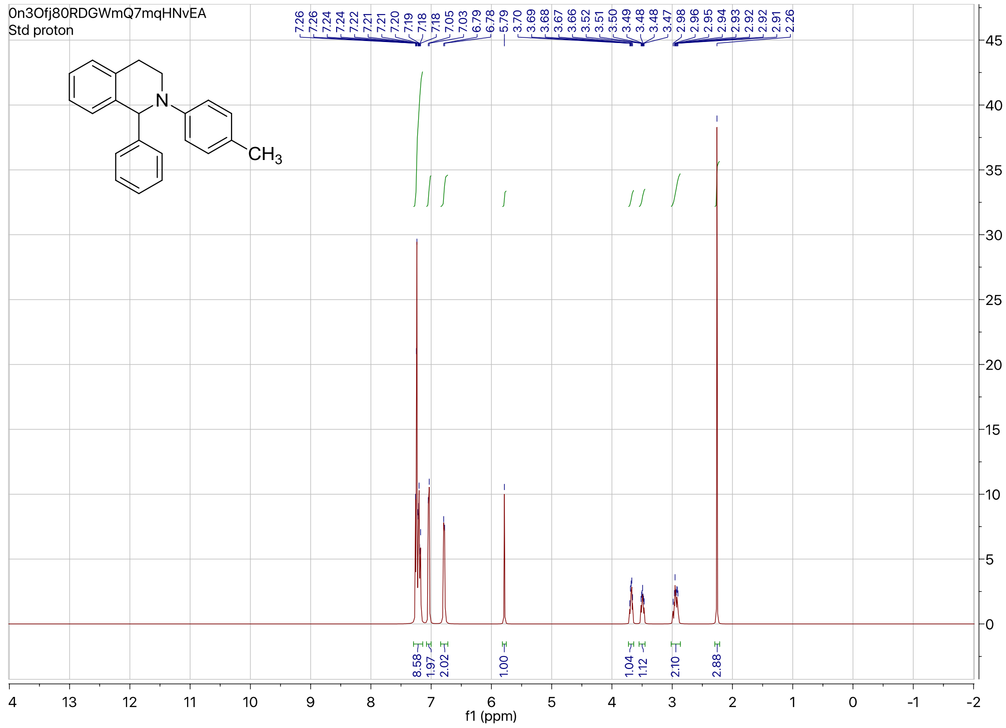
0n3Ofj80RDGWmQ7mqHNvEA
Std proton
N
CH3
f1 (ppm)

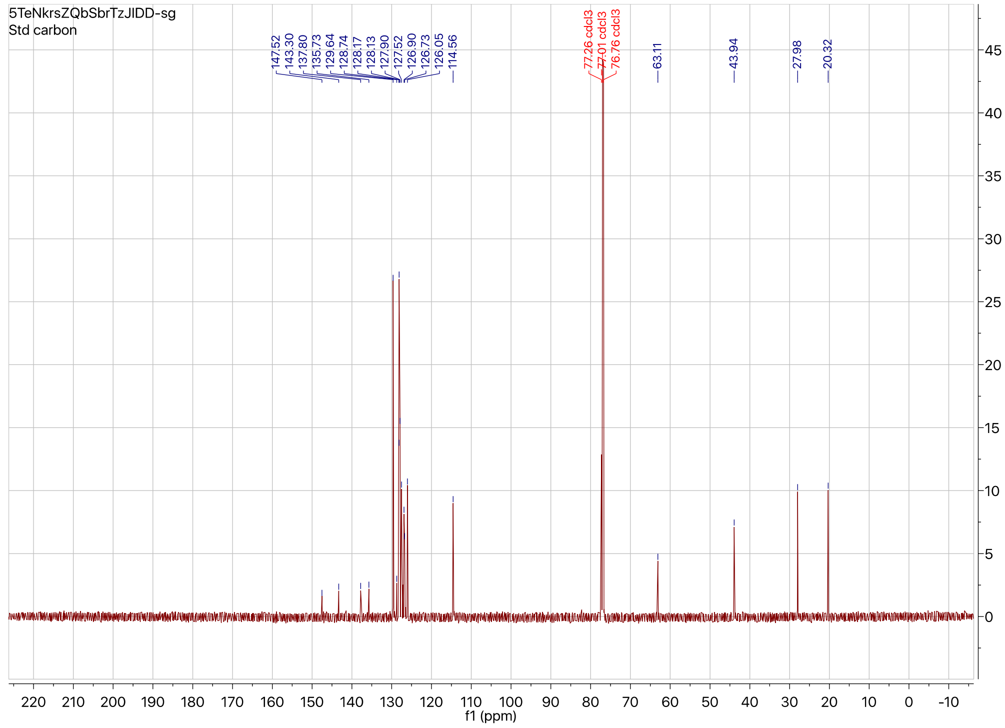
5TeNkrsZQbSbrTzJIDD-sg
Std carbon
f1 (ppm)

3f

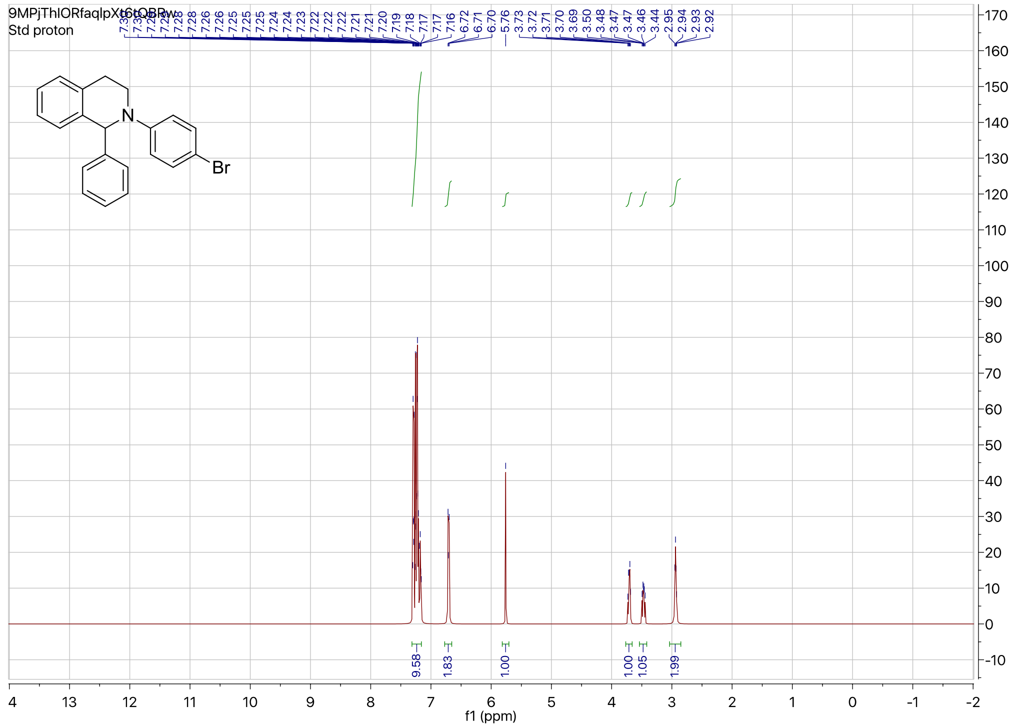

9MPjThlORfaqlpXt6tQBRw
Std proton
N
Br
7.28
7.26
7.26
7.25
7.25
7.25
7.24
7.24
7.23
7.22
7.22
7.22
7.22
7.21
7.21
7.20
7.19
7.18
7.17
7.17
7.16
6.72
6.71
6.70
5.76
3.73
3.72
3.71
3.70
3.69
3.50
3.48
3.47
3.47
3.46
3.44
2.95
2.94
2.93
2.92
9.58
1.83
1.00
1.00
1.05
1.99
f1 (ppm)

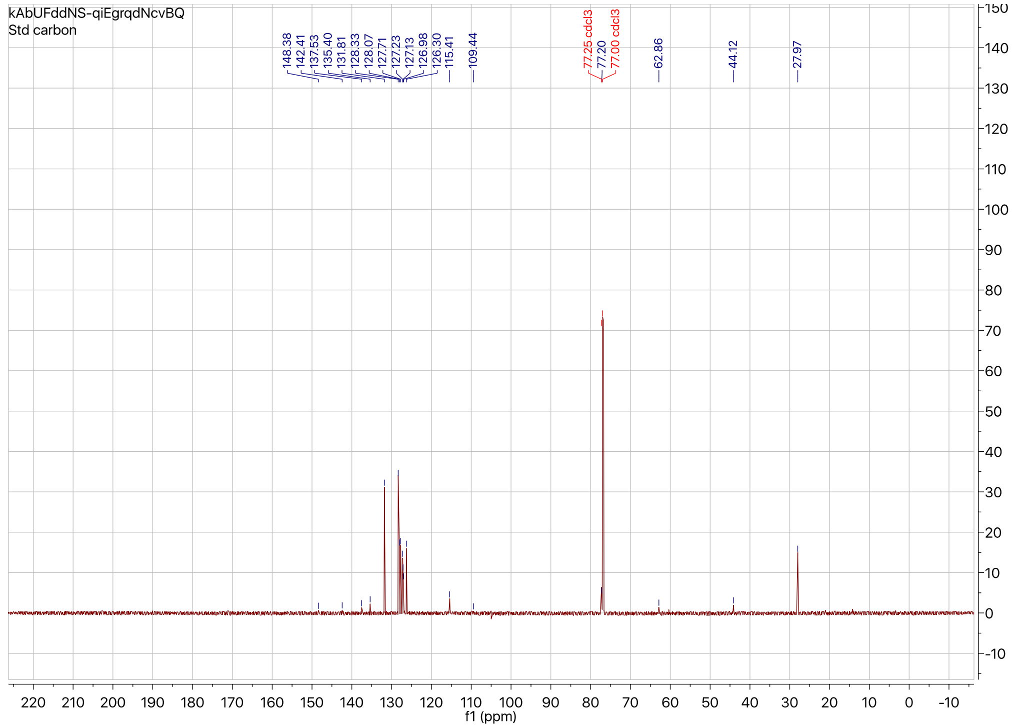

kAbUFddNS-qiEgrqdNcvBQ
Std carbon
148.38
142.41
137.53
135.40
131.81
128.33
128.07
127.71
127.23
127.13
126.98
126.30
115.41
109.44
77.25 cdcl3
77.20
77.00 cdcl3
62.86
44.12
27.97
f1 (ppm)

3g

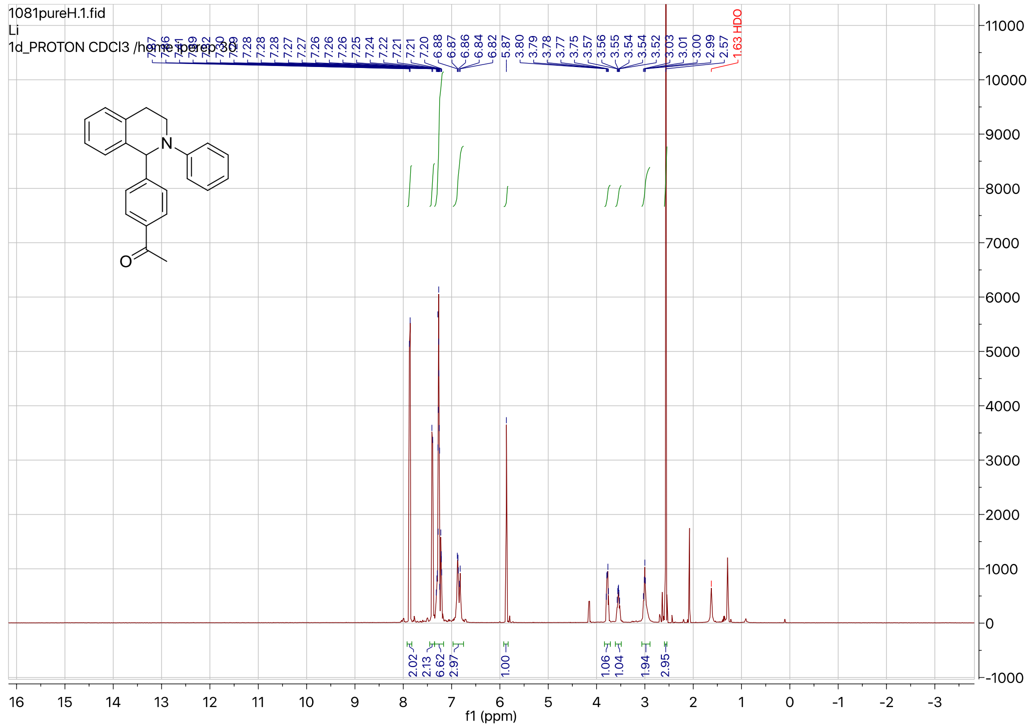

1081pureH.1.fid
Li
1d_PROTON CDCl3 /home iperep 30
1.63 HDO
f1 (ppm)

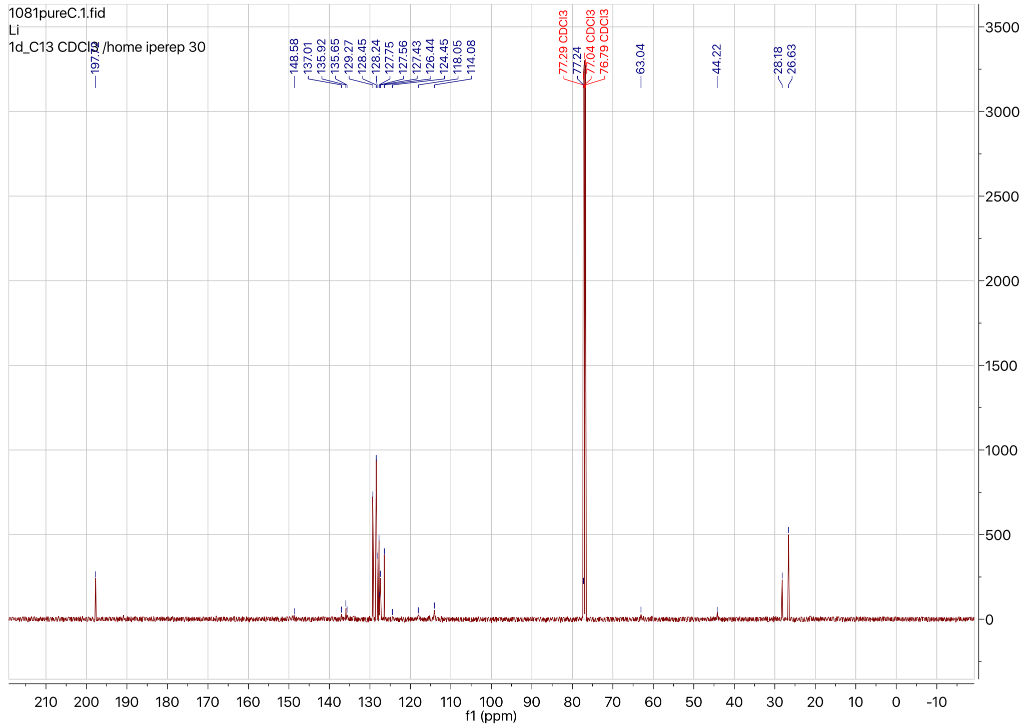

1081pureC.1.fid
Li
1d_C13 CDCl3 /home iperep 30
148.58
137.01
135.92
135.65
129.27
128.45
128.24
127.75
127.56
127.43
126.44
124.45
118.05
114.08
77.29 CDCl3
77.24
77.04 CDCl3
76.79 CDCl3
63.04
44.22
28.18
26.63
f1 (ppm)

3h

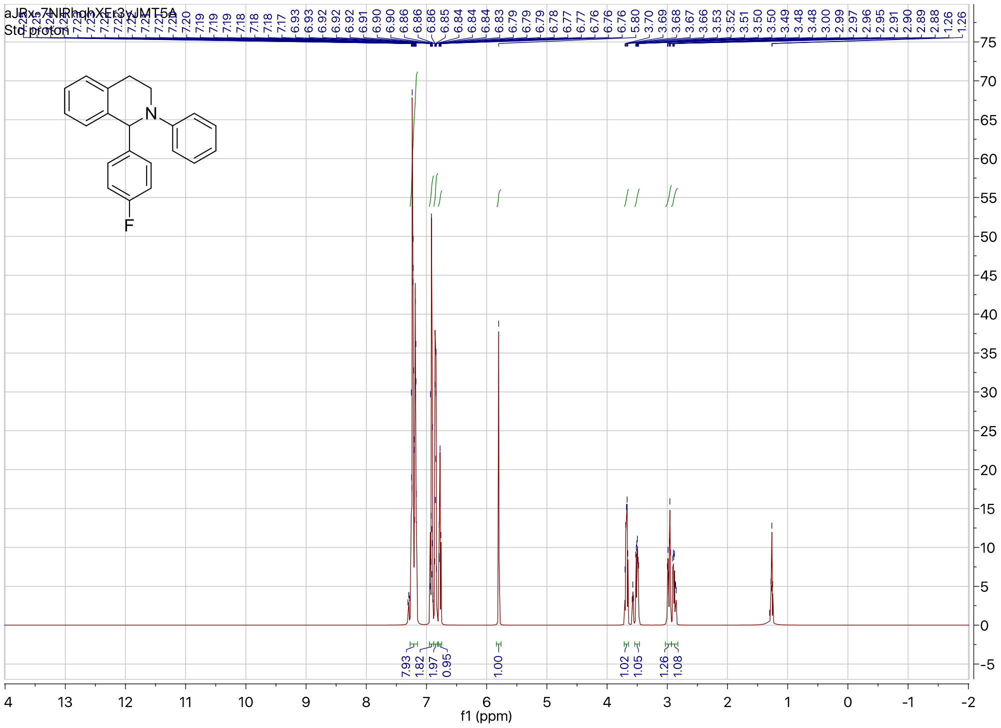
aJRx-7NIPhqhXEr3yJMT5A
Std proton
N
F
f1 (ppm)
7.93
1.82
1.97
0.95
1.00
1.02
1.05
1.26
1.08

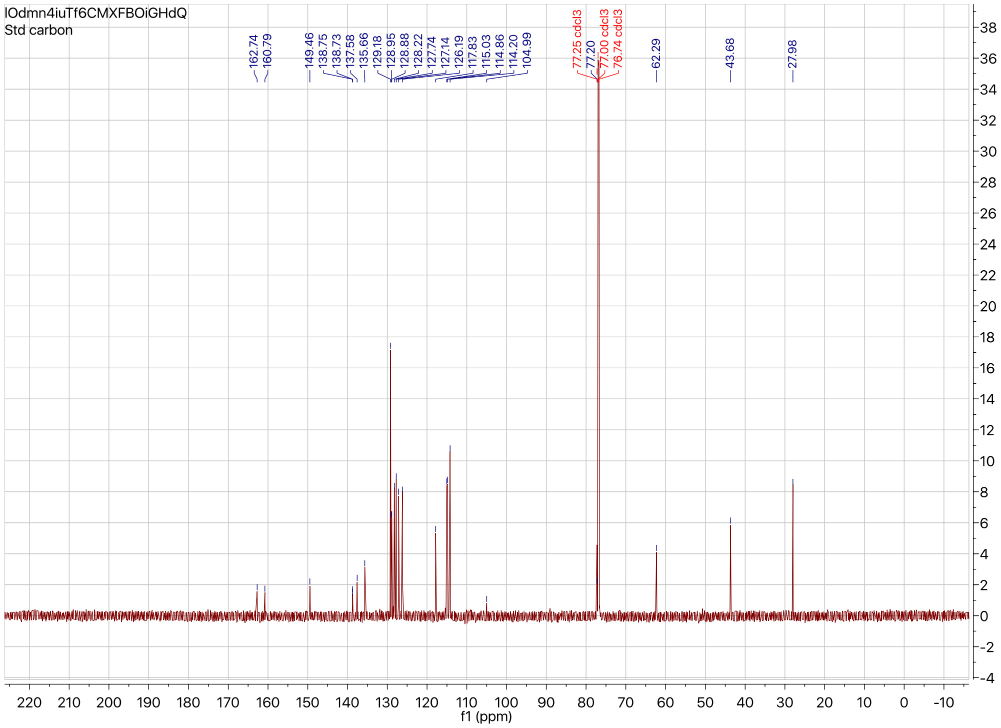
lOdmn4iuTf6CMXFBOiGHdQ
Std carbon
162.74
160.79
149.46
138.75
138.73
137.58
135.66
129.18
128.95
128.88
128.22
127.74
127.14
126.19
117.83
115.03
114.86
114.20
104.99
77.25 cdcl3
77.20
77.00 cdcl3
76.74 cdcl3
62.29
43.68
27.98
f1 (ppm)

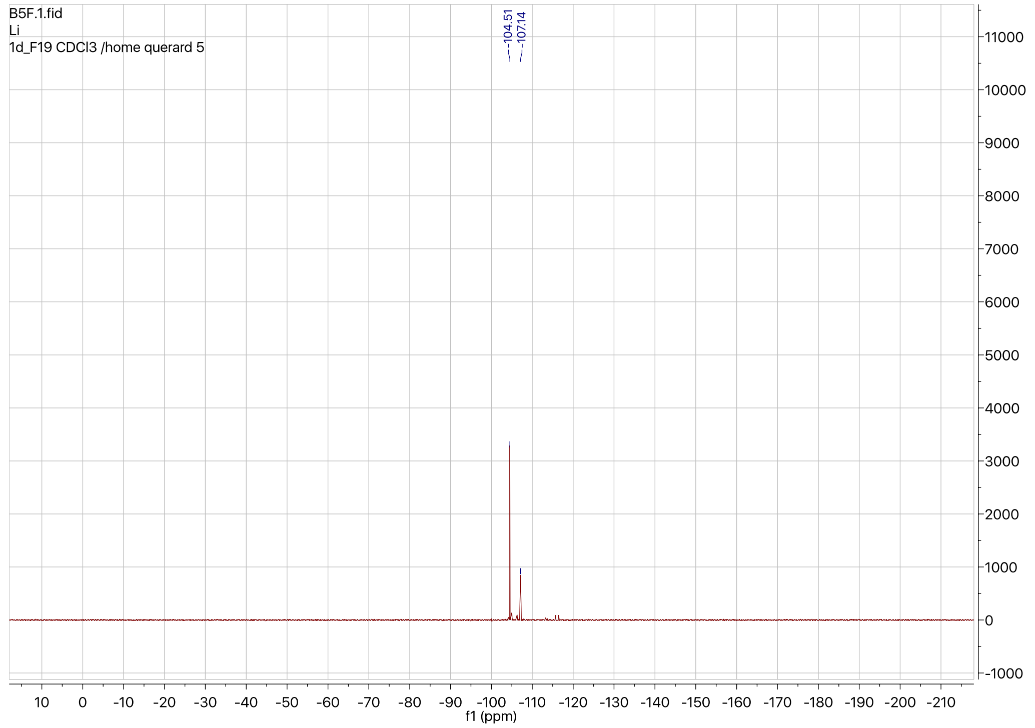

B5F.1.fid
Li
1d_F19 CDCl3 /home querard 5
-104.51
-107.14
11000
10000
9000
8000
7000
6000
5000
4000
3000
2000
1000
0
-1000
10 0 -10 -20 -30 -40 -50 -60 -70 -80 -90 -100 -110 -120 -130 -140 -150 -160 -170 -180 -190 -200 -210
f1 (ppm)

3i

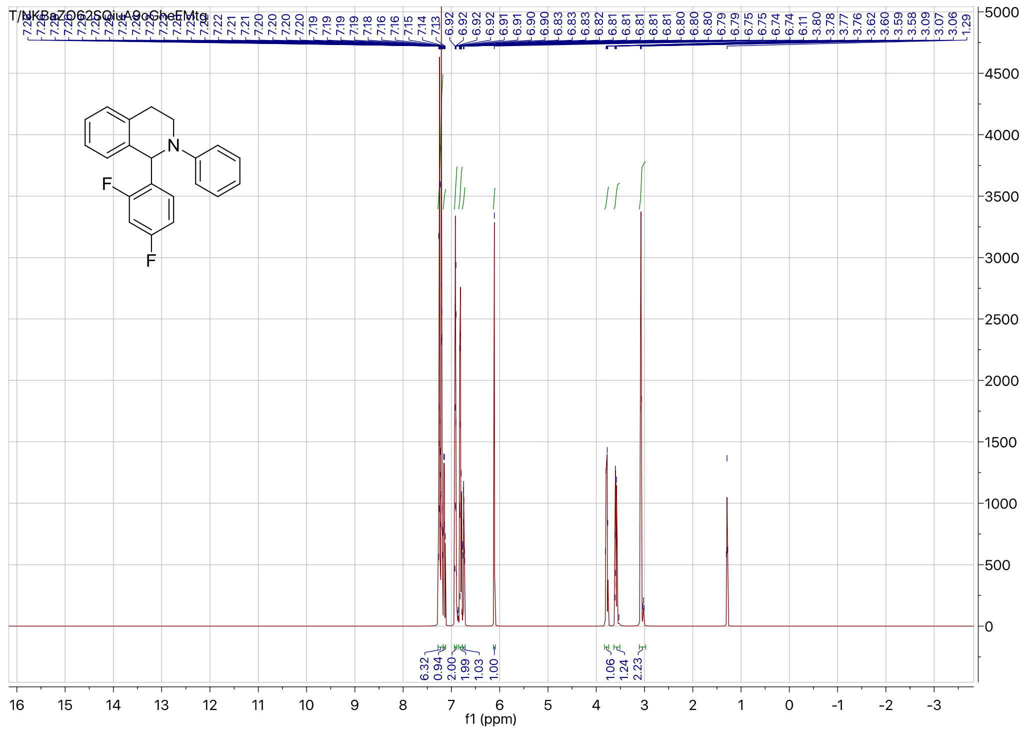
f1 (ppm)
6.32
0.94
2.00
1.99
1.03
1.00
1.06
1.24
2.23

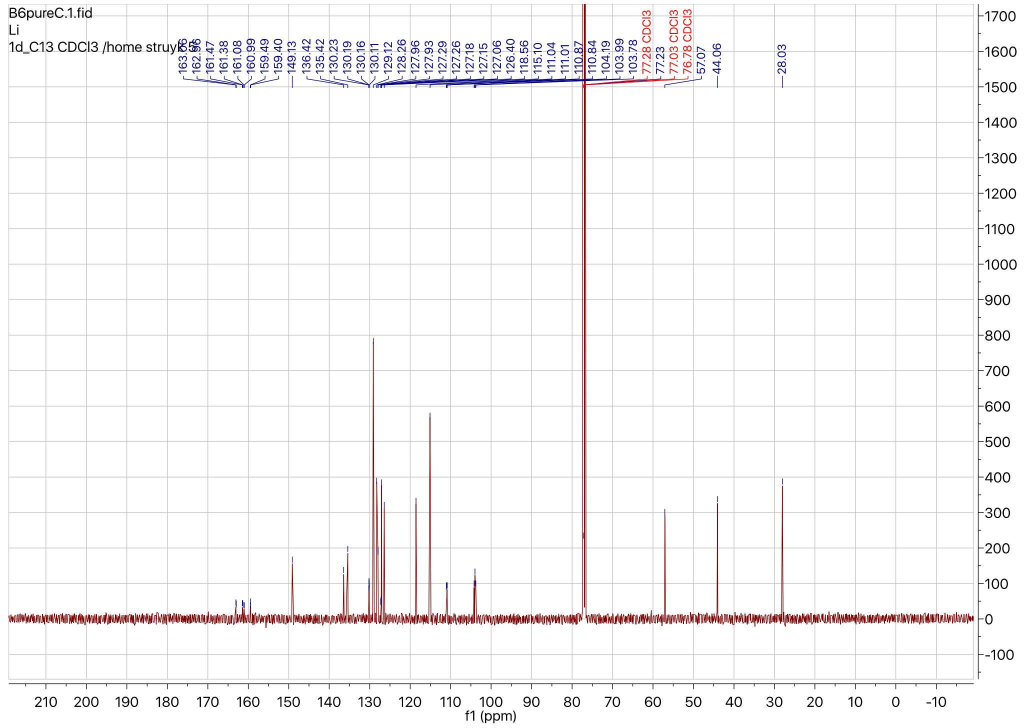
B6pureC.1.fid
Li
1d_C13 CDCl3 /home struy
149.13
136.42
135.42
130.23
130.19
130.16
130.11
129.12
128.26
127.96
127.93
127.29
127.26
127.18
127.15
127.06
126.40
118.56
115.10
111.04
111.01
110.87
110.84
104.19
103.99
103.78
77.28 CDCl3
77.23
77.03 CDCl3
76.78 CDCl3
57.07
44.06
28.03
f1 (ppm)

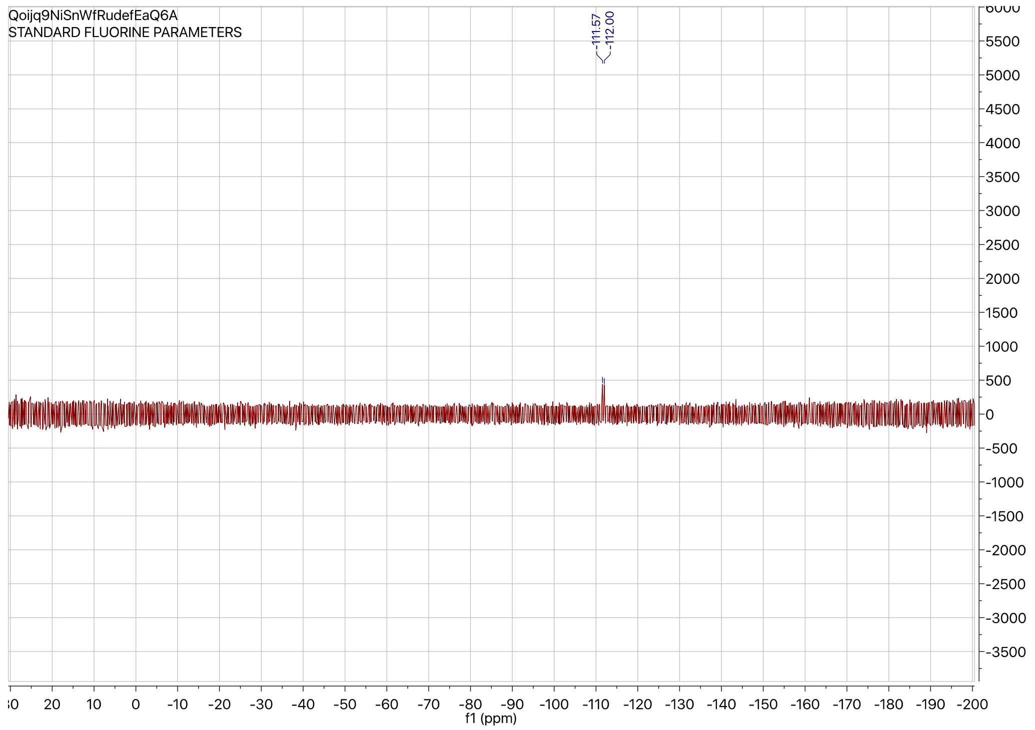

Qoijq9NiSnWfRudefEaQ6A
STANDARD FLUORINE PARAMETERS
-111.57
-112.00
f1 (ppm)

3j

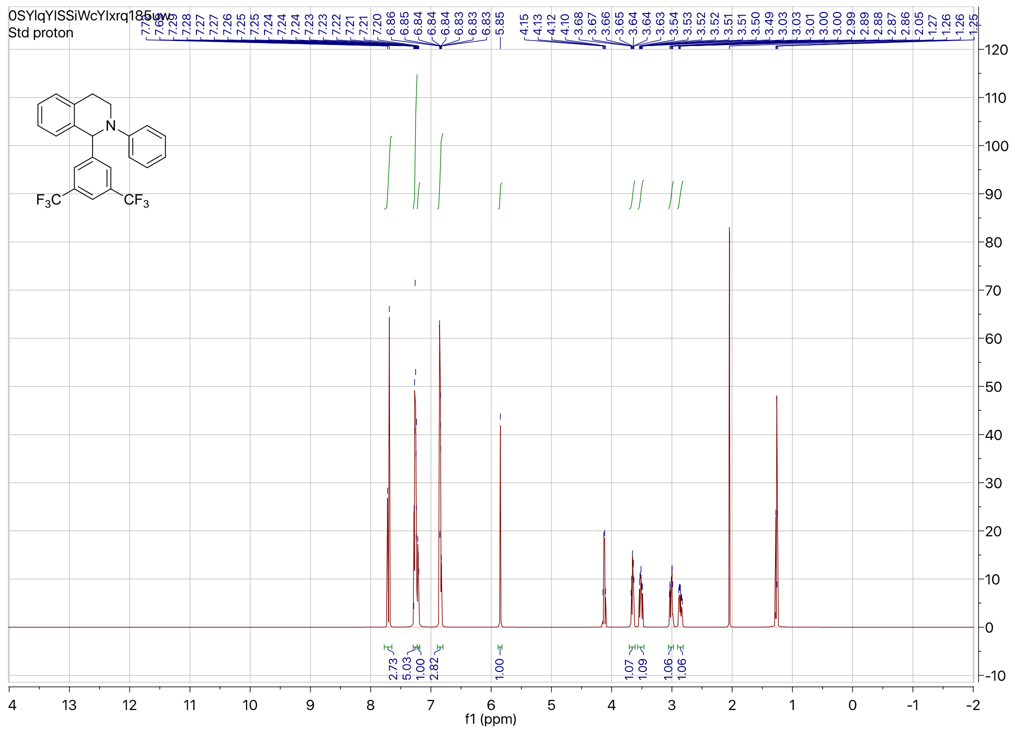

0SYlqYlSSiWcYlxrq185uw
Std proton
F$_3$C
CF$_3$
N
f1 (ppm)

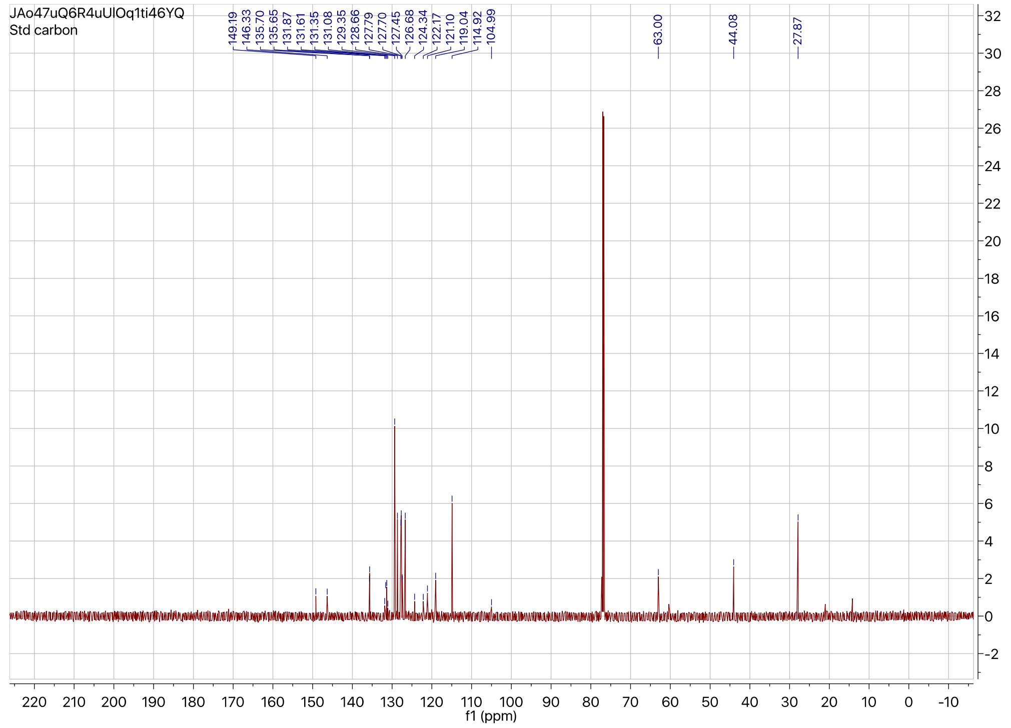

JAo47uQ6R4uUlOq1ti46YQ
Std carbon
149.19
146.33
135.70
135.65
131.87
131.61
131.35
131.08
129.35
128.66
127.79
127.70
127.45
126.68
124.34
122.17
121.10
119.04
114.92
104.99
63.00
44.08
27.87
f1 (ppm)

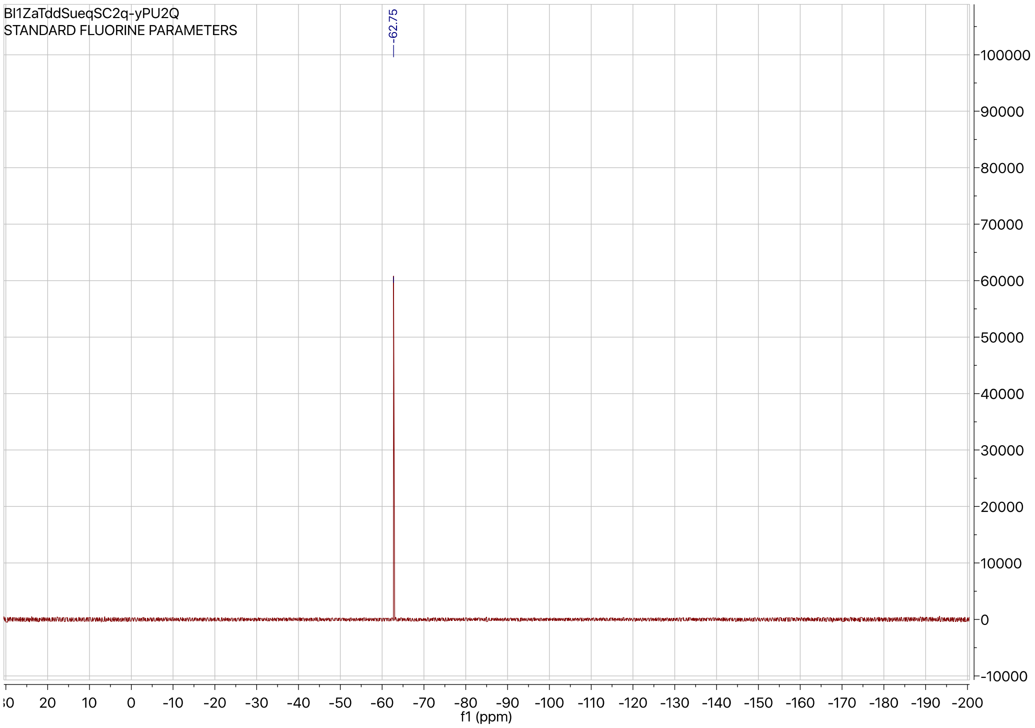

Bl1ZaTddSueqSC2q-yPU2Q
STANDARD FLUORINE PARAMETERS
-62.75
f1 (ppm)

3k

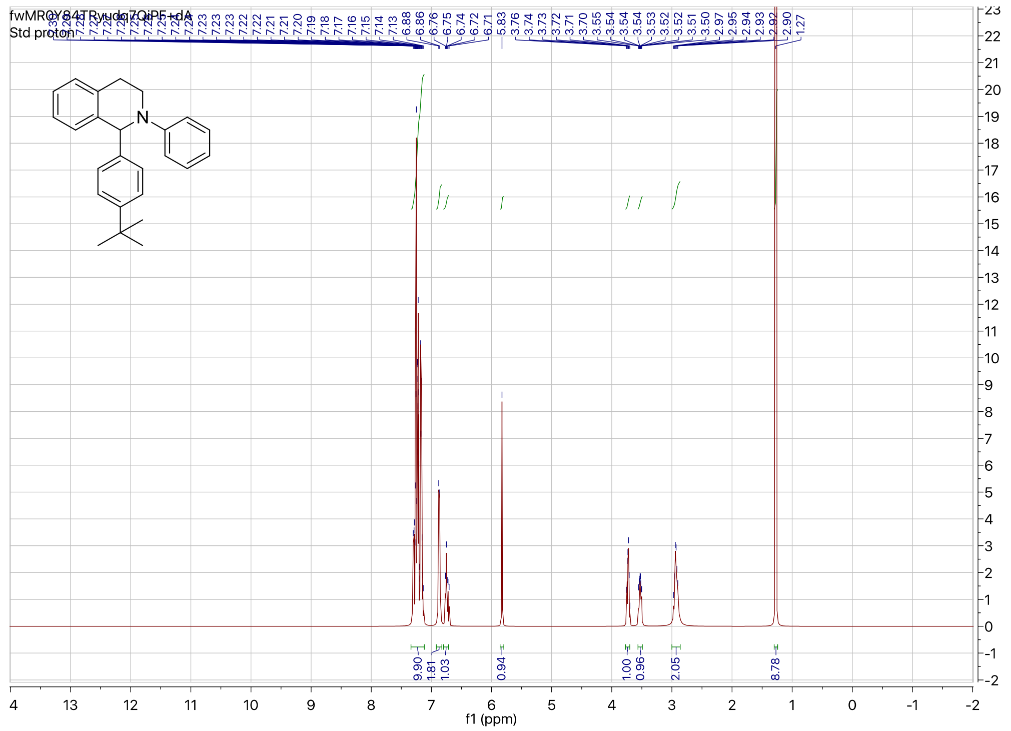
Std proton
N
f1 (ppm)
5.83
1.27
9.90
1.81
1.03
0.94
1.00
0.96
2.05
8.78

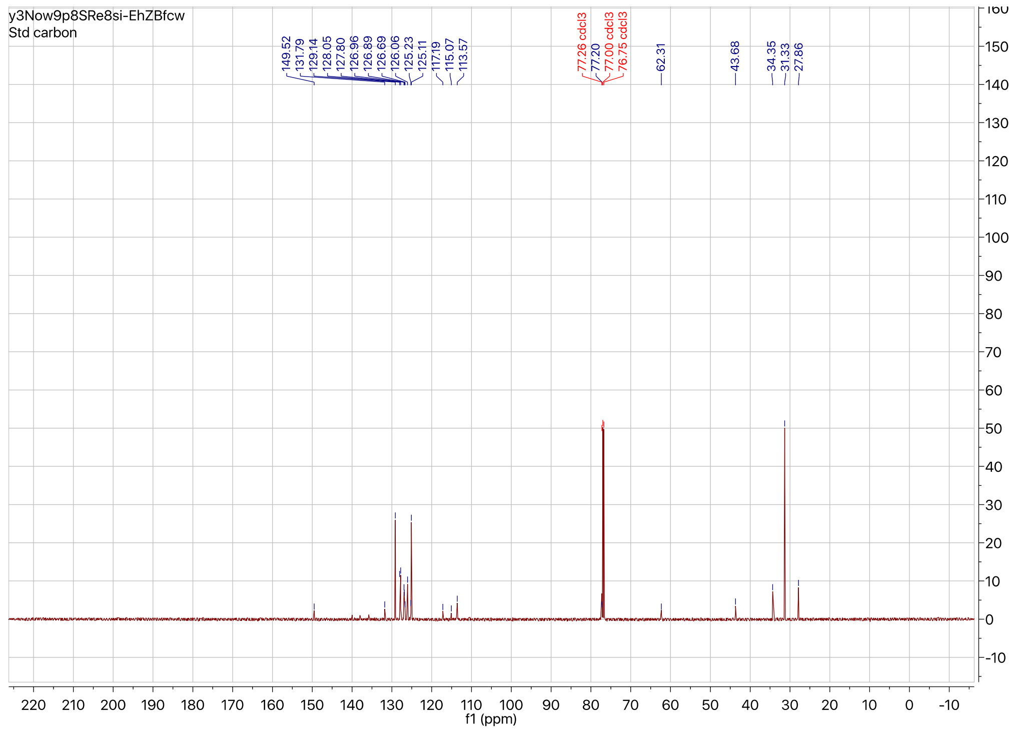
y3Now9p8SRe8si-EhZBfcw
Std carbon
149.52
131.79
129.14
128.05
127.80
126.96
126.89
126.69
126.06
125.23
125.11
117.19
115.07
113.57
77.26 cdcl3
77.20
77.00 cdcl3
76.75 cdcl3
62.31
43.68
34.35
31.33
27.86
f1 (ppm)

3l

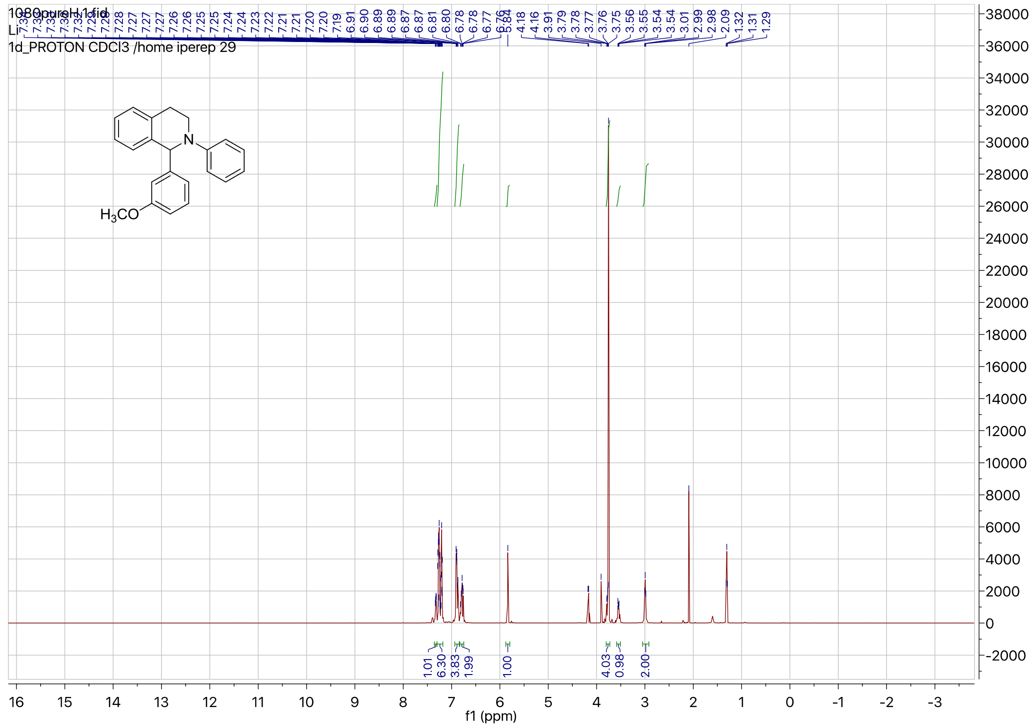
1080pureH.1.fid
Li
1d_PROTON CDCl3 /home iperep 29
7.28 7.27 7.27 7.27 7.26 7.26 7.25 7.25 7.24 7.24 7.23 7.22 7.21 7.21 7.20 7.20 7.19 6.91 6.90 6.89 6.89 6.87 6.87 6.81 6.80 6.78 6.78 6.77 5.84 4.18 4.16 3.91 3.79 3.78 3.77 3.76 3.75 3.56 3.55 3.54 3.54 3.01 2.99 2.98 2.09 1.32 1.31 1.29
N
H_3CO
1.01 6.30 3.83 1.99 1.00 4.03 0.98 2.00
f1 (ppm)

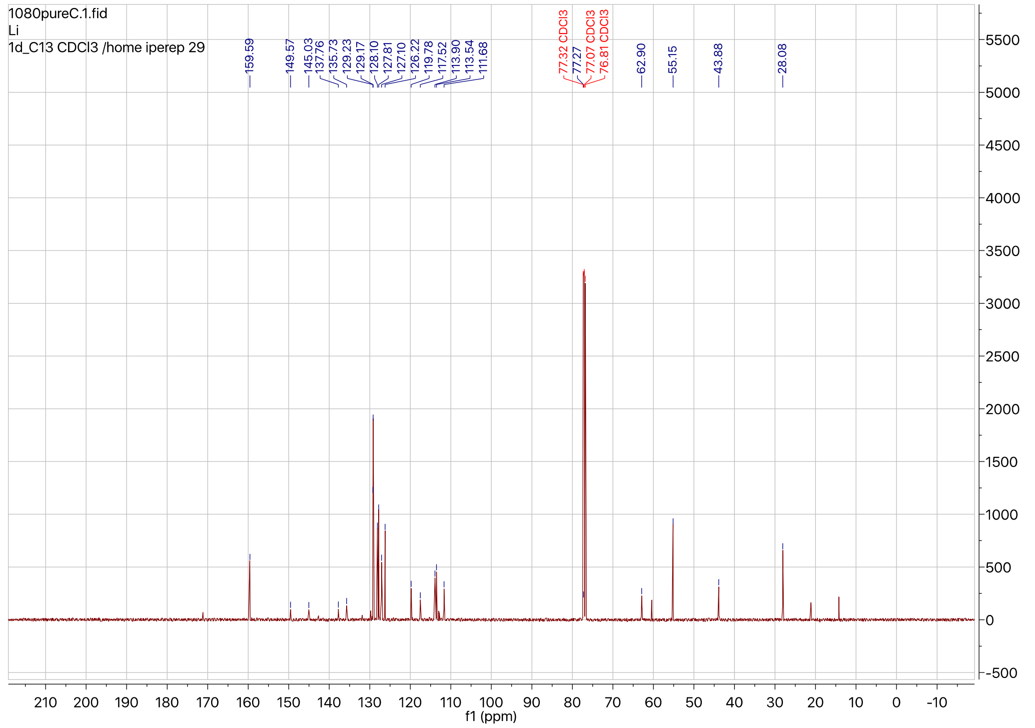
1080pureC.1.fid
Li
1d_C13 CDCl3 /home iperep 29
159.59 149.57 145.03 137.76 135.73 129.23 129.17 128.10 127.81 127.10 126.22 119.78 117.52 113.90 113.54 111.68
77.32 CDCl3 77.27 77.07 CDCl3 76.81 CDCl3
62.90 55.15 43.88 28.08
f1 (ppm)

3m

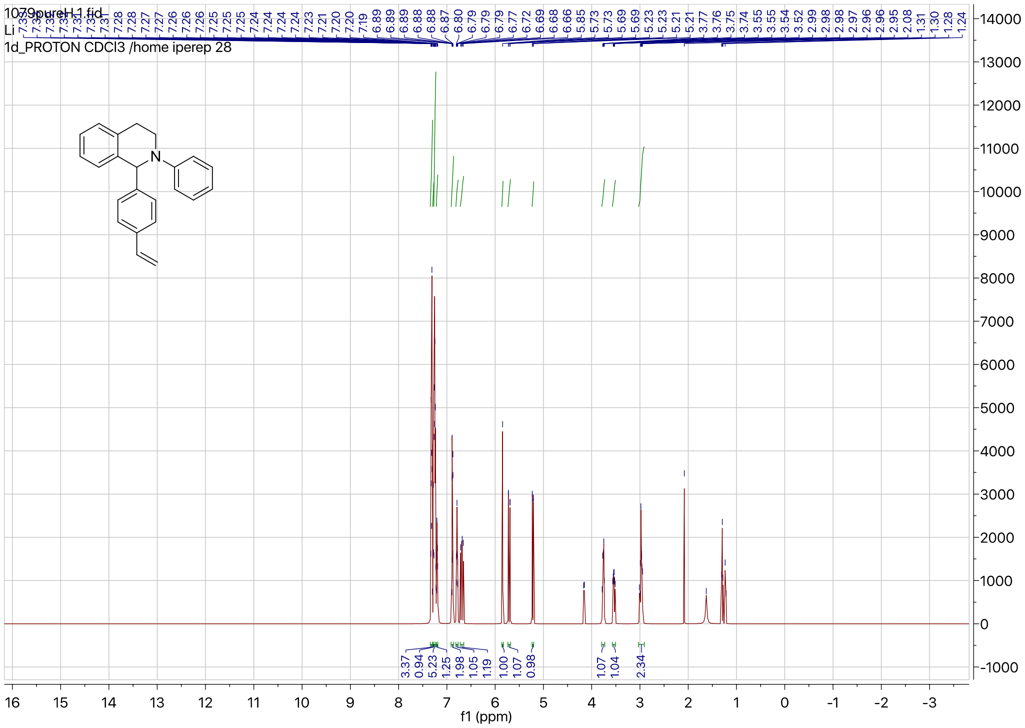
1079pureH.1.fid
Li
1d_PROTON CDCl3 /home iperep 28
N
3.37
0.94
5.23
1.25
1.98
1.05
1.19
1.00
1.07
0.98
1.07
1.04
2.34
f1 (ppm)

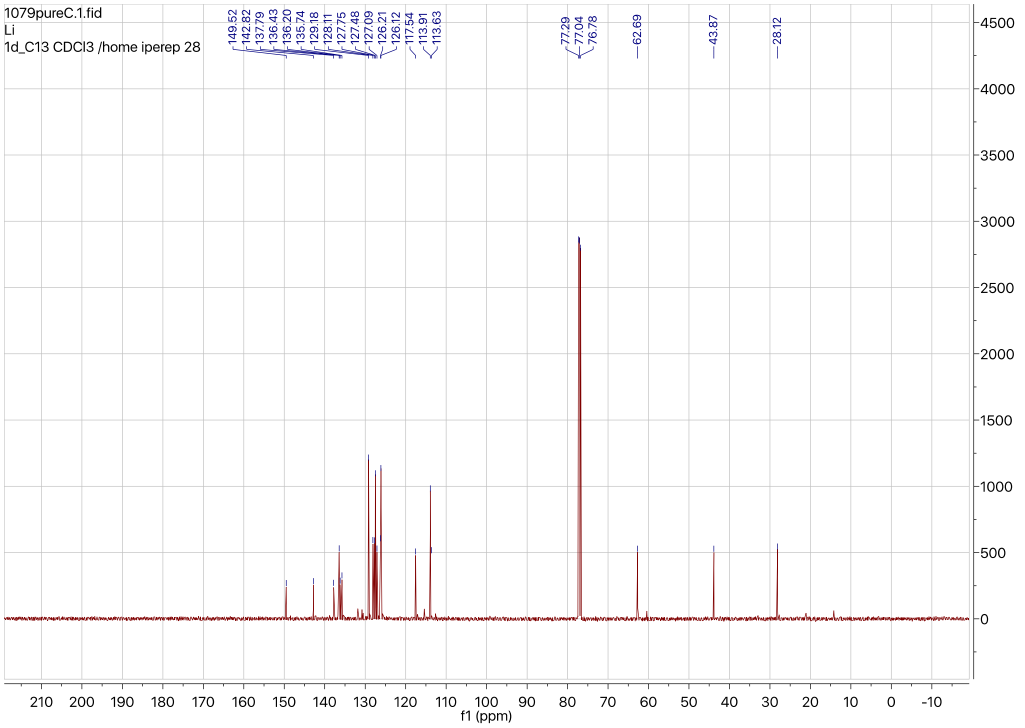
1079pureC.1.fid
Li
1d_C13 CDCl3 /home iperep 28
149.52
142.82
137.79
136.43
136.20
135.74
129.18
128.11
127.75
127.48
127.09
126.21
126.12
117.54
113.91
113.63
77.29
77.04
76.78
62.69
43.87
28.12
f1 (ppm)

3n

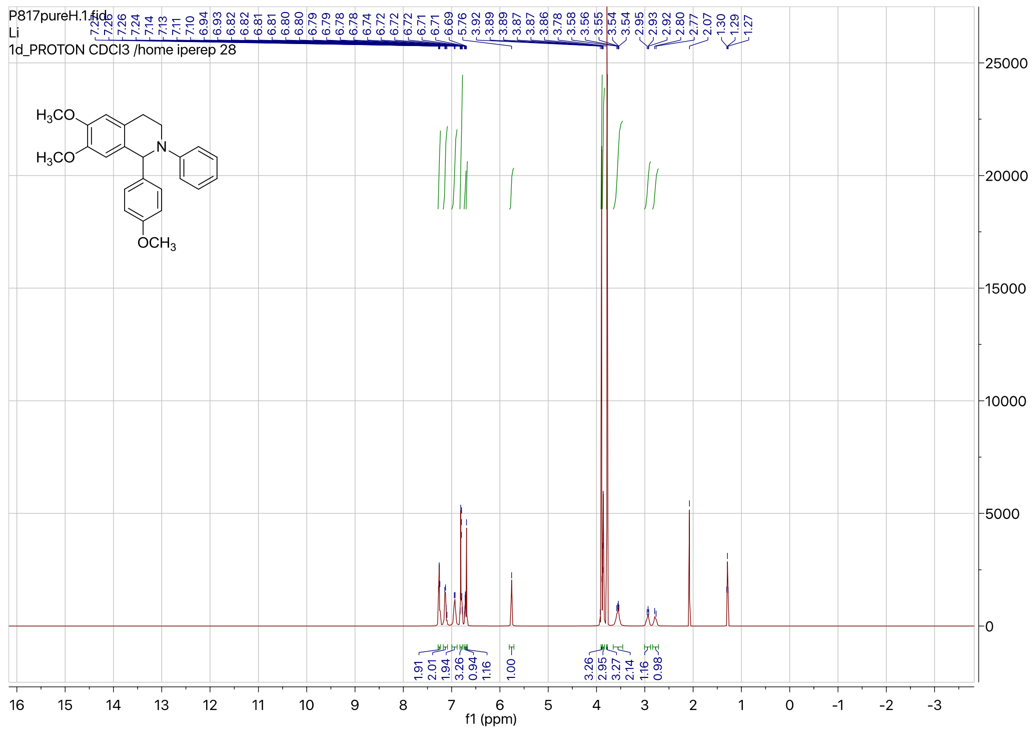
P817pureH.1.fid
Li
1d_PROTON CDCl3 /home iperep 28
H3CO
H3CO
N
OCH3
f1 (ppm)

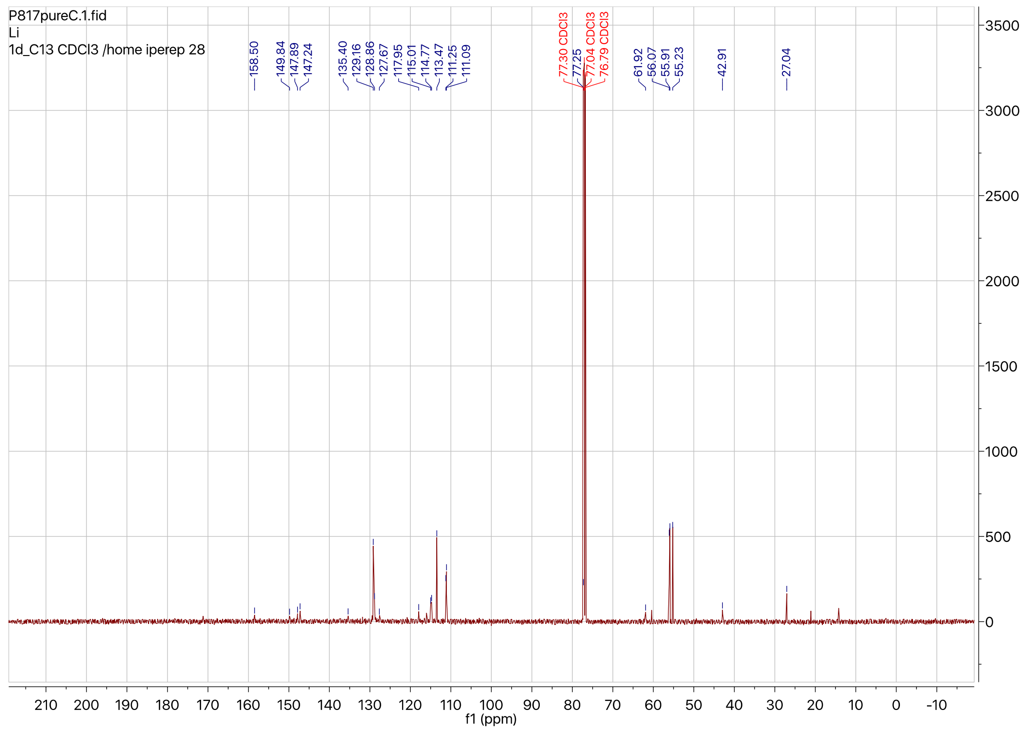
P817pureC.1.fid
Li
1d_C13 CDCl3 /home iperep 28
158.50
149.84
147.89
147.24
135.40
129.16
128.86
127.67
117.95
115.01
114.77
113.47
111.25
111.09
77.30 CDCl3
77.25
77.04 CDCl3
76.79 CDCl3
61.92
56.07
55.91
55.23
42.91
27.04
f1 (ppm)

[1] Zhiping Li, Z.; MacLeod, P. D.; Li, C.-J. *Tetrahedron Lett*. **2002**, *43*, 5731

[2] Rueping, M.; Koenigs, R. M.; Poscharny, K.; Fabry, D. C.; Leonori,D.; Carlos Vila, C. *Chem. Eur. J.* **2012**, 18, 5170

[3] Kwong, F. Y.; Klapars, A.; Buchwald, S. L. *Org. Lett.* **2002**, *4*, 581

[4] Slinker, J. D.; Gorodetsky, A. A.; Lowry, M. S.; Wang, J.; Parker, S.; Rohl, R.; Bernhard, S.; Malliaras, G. G. *J. Am. Chem. Soc.* **2004**, *126*, 2763

[5] Sprouse, S.; K. A. King, K. A.; P. J. Spellane, P. J.; Watts, R. J. *J. Am. Chem. Soc***. 1984,** *106,* 6647

[6] Zeng, T.; Song, G.; Moores, A.; Li, C.-J. *Synlett.* **2010**, *13*, 2002

[7] Nauth, A. M.; Otto, N.; Opatz, T. *Adv. Synth. Catal.* **2015**, *357*, 3424
